# Designing for empowerment impact in agricultural development projects: Experimental evidence from the Agriculture, Nutrition, and Gender Linkages (ANGeL) project in Bangladesh

**DOI:** 10.1016/j.worlddev.2021.105622

**Published:** 2021-10

**Authors:** Agnes Quisumbing, Akhter Ahmed, John Hoddinott, Audrey Pereira, Shalini Roy

**Affiliations:** a International Food Policy Research Institute, Washington, DC, United States; b Cornell University, Ithaca, NY, United States; c University of North Carolina, Chapel Hill, NC, United States

**Keywords:** Women’s empowerment, Gender norms, Nutrition-sensitive agriculture, Randomized controlled trial, Asia, Bangladesh

## Abstract

The importance of women’s roles for nutrition-sensitive agricultural projects is increasingly recognized, yet little is known about whether such projects improve women’s empowerment and gender equality. We study the Agriculture, Nutrition, and Gender Linkages (ANGeL) pilot project, which was implemented as a cluster-randomized controlled trial by the Government of Bangladesh. The project’s treatment arms included agricultural training, nutrition behavior change communication (BCC), and gender sensitization trainings delivered to husbands and wives together – with these components combined additively, such that the impact of gender sensitization could be distinguished from that of agriculture and nutrition trainings. Empowerment was measured using the internationally-validated project-level Women’s Empowerment in Agriculture Index (pro-WEAI), and attitudes regarding gender roles were elicited from both men and women, to explore potentially gender-transformative impacts. Our study finds that ANGeL increased both women’s and men’s empowerment, raised the prevalence of households achieving gender parity, and led to small improvements in the gender attitudes of both women and men. We find significant increases in women’s empowerment scores and empowerment status from all treatment arms but with no significant differences across these. We find no evidence of unintended impacts on workloads and inconclusive evidence around impacts on intimate partner violence. Our results also suggest some potential benefits of bundling nutrition and gender components with an agricultural development intervention; however, many of these benefits seem to be driven by bundling nutrition with agriculture. While we cannot assess the extent to which including men and women within the same treatment arms contributed to our results, it is plausible that the positive impacts of all treatment arms on women’s empowerment outcomes may have arisen from implementation modalities that provided information to both husbands and wives when they were together. The role of engaging men and women jointly in interventions is a promising area for future research.

## Highlights

• Using an RCT, we assess the impacts of agriculture, nutrition, and gender interventions on women’s empowerment in Bangladesh.
• Single or bundled trainings on agriculture, nutrition, and gender were provided to husbands and wives jointly.
• All interventions improved women’s empowerment without disempowering men.
• Nutrition trainings improved men’s gender attitudes, particularly on women’s responsibilities around cooking and childcare.
• The role of engaging men and women jointly in interventions is a promising area for future research.

## 1 Introduction

The literature on intrahousehold allocation has long recognized the important role of women in attaining good health and nutritional status of their household members (Thomas, 1990; Haddad & Hoddinott, 1994). Similarly, women play a key role in the linkages between agriculture and nutrition (Ruel & Alderman, 2013; Kadiyala, Harris, Headey, Yosef, & Gillespie, 2014). In the framework proposed by Ruel and Alderman (2013), three of the six agriculture-nutrition pathways focus on women’s roles, and one pathway specifically identifies women’s status and empowerment as a key determinant of nutrition. The lessons emerging from this literature have influenced the design and implementation of nutrition-sensitive agricultural projects (Ruel, Quisumbing, & Balagamwala, 2018). Increasingly, such projects have gone beyond focusing only on women, deliberately including programming that recognizes women’s and men’s different roles in providing for their households’ food security and nutrition (Malapit et al., 2019). However, the extent to which these projects empower women and improve gender equality remains unclear.

This evidence base is weak for several reasons. First, although many project designers aim to empower women, most do not distinguish whether their projects do so, or simply reach (i.e. encourage participation) and benefit them (Johnson et al., 2018). While “reach” and “benefit” are frequently evaluated, whether these projects have empowered women often remains unaddressed. Moreover, proponents of gender-transformative approaches (Wong, Vos, Pyburn, & Newton, 2019) argue that focusing only on factors affecting an individual woman’s empowerment, without aspiring to transform gender norms, fails to address the foundations of gender inequity and unequal power relations. These gender-transformative aspects are often not assessed in research on agricultural development projects.

Second, measurement issues make it difficult to assess whether defined objectives relating to empowerment are achieved. Even if projects aim to empower women or transform gender norms, the absence of a common quantitative metric for women’s empowerment makes it difficult to evaluate the impact of these projects in a comparable manner (Brody et al., 2017). Although qualitative studies have yielded valuable insights into what aspects of these projects empower women, generalizing these findings beyond their specific context to inform decisions about adapting and scaling up programs is challenging. In addition, many studies use measures that rely only on data collected from women, making it impossible to ascertain the impact on men. For many objectives related to empowerment – such as changing gender norms – assessing effects on men is critical. Moreover, little is known about whether increases in women’s empowerment occur at the expense of men’s empowerment, which cannot be assessed if men’s empowerment is not measured.

Third, additional challenges relate to understanding the role of program design. Agricultural projects that account for gender relations often engage only with women. It is likely that engaging men may also be important to affect empowerment outcomes; here too, however, the evidence base is thin. Moreover, many nutrition-sensitive agricultural projects that also have gender-related objectives implement bundled strategies. This makes it difficult to tease out the differential or additive effect of strategies that aim to empower women or change gender norms (Ruel, Quisumbing, & Balagamwala, 2018). A specific knowledge gap exists on the extent to which women’s involvement in agricultural projects alone can improve their empowerment relative to additional programming, and to what extent additional programming needs to be aimed specifically at improving gender relations.

This paper seeks to redress these gaps by providing evidence from a randomized controlled trial (RCT) of a gender- and nutrition-sensitive agricultural project in Bangladesh, the Agriculture, Nutrition, and Gender Linkages (ANGeL) project. ANGeL’s treatment arms provided agricultural training, nutrition behavior change communication (BCC), and gender sensitization trainings to husbands and wives together, thus engaging men alongside women. The randomly assigned treatments were designed to be additive, allowing the impact of additional nutrition and gender sensitization trainings to be distinguished from the impact of agricultural training. We assess impact using the project-level Women’s Empowerment in Agriculture Index (Pro-WEAI) (Malapit et al., 2019). Pro-WEAI is based on information collected from both women and men within a household, allowing both men’s and women’s empowerment to be assessed. We assess the impact of ANGeL’s treatment arms on (1) women’s and men’s empowerment, as well as household gender parity; (2) women’s and men’s attitudes towards gender norms (that is, were impacts gender transformative); and (3) unintended consequences, namely changes in women’s and men’s workloads or changes in intimate partner violence (IPV).

## 2 Study background and motivation

The ANGeL pilot project was motivated in part by high rates of child and maternal undernutrition in Bangladesh (NIPORT (National Institute of Population Research and Training), 2015, icddr, b (International Center for Diarrheal Disease Research, Bangladesh), UNICEF (UN Children’s Fund), GAIN (Global Alliance for Improved Nutrition), IPHN (Institute of Public Health and Nutrition), 2013) despite decades of progress in domestic food production. Evidence from Bangladesh suggests agriculture has the potential to be a driver of nutrition (e.g., Heady & Hoddinott, 2016), but that this potential is not fully realized when nutrition and agricultural policies are not coordinated or when gender inequities prevail. In terms of linkages in nutrition and agriculture policies, studies from Bangladesh show that coupling increases in income with nutrition training leads to greater improvements in child nutrition than increasing income alone, in part through diversifying diets (Ahmed et al., 2019). In terms of gender, low status of women and gender gaps in health and education are found to contribute to chronic child undernutrition (Smith et al., 2003) and food insecurity (von Grebmer, 2009), even with improvements in income. In Bangladesh, women are key actors within the food system, but are historically disempowered in terms of leadership in the community, control of resources, and control of income (Sraboni, Quisumbing, & Ahmed, 2014). Despite increases in women’s participation in agriculture in Bangladesh (Asaduzzaman, 2010), women continue to have limited control over agricultural assets, as well as limited mobility to go to markets to sell agricultural produce. Representative data from rural Bangladesh (Sraboni, Malapit, Quisumbing, & Ahmed, 2014) show agricultural production diversity is associated with dietary diversity, and women’s empowerment is associated with dietary diversity as well, indicating that women’s disempowerment could weaken the links between agriculture and nutrition. Together, these observations form the rationale for testing how trainings in agriculture and nutrition – separately and additively – affect nutrition outcomes, as well as how added gender trainings shape these effects. This is one focus of the overall ANGeL evaluation, explored in a companion paper.

The questions, however, can also be reframed with a view of empowerment as an intrinsic objective: How do trainings in agriculture and nutrition – separately and additively – affect empowerment outcomes, and how do added gender trainings shape these empowerment effects? This is another focus of the ANGeL evaluation and where we concentrate in the present paper. Evidence suggests that trainings may empower participants even if empowerment is not their primary objective. In Bangladesh, a nutrition training added to food or cash transfers was found to not only improve nutrition outcomes more than the transfers alone (Ahmed et al., 2019, Hoddinott et al., 2018), but also to cause long-lasting improvements in dimensions of women’s empowerment and reductions in intimate partner violence (IPV) that persisted four years post-program (Roy et al., 2019, Roy et al., 2019). These sustained improvements were hypothesized to occur in part due to increases in participants’ feelings of agency and social capital (from interaction with other training participants, as well as greater respect within the community because of participants’ increased knowledge). A systematic review of women’s self-help groups by Brody et al. (2017) and a review of mechanisms operating in women’s groups by Dìaz-Martin, Gopalan, Guarnieri, and Jayachandran (2020) also indicate that bringing together group participants, particularly women, could empower them. Moreover, it is plausible that inviting men and women in the same household together for training on the same topic could increase joint decisionmaking around that topic; this may itself be empowering, given qualitative evidence from Bangladesh that individuals tend to view empowerment as communal and focused on the family unit (rather than singular and focused on the individual woman or man), and perceive it as including the ability to work jointly and well together (Alkire et al., 2013). Potentially, training on a topic could be most empowering for participants with limited engagement in that topic prior to training, thus empowerment impacts by gender could possibly differ based on whether the topic is traditionally dominated by men or women. Together, these observations suggest plausible mechanisms for trainings on agriculture or nutrition to increase empowerment for husbands and/or wives – as well as the possibility that effects could differ by topic, and that bundling training for the two topics could change the effects (e.g., due to a greater number of topics or training sessions). At the same time, Diaz-Martin et al. (2020) find evidence that “intentional program design” that delivers content directly related to gender-specific issues may be important for changing outcomes such as decision-making, self-efficacy, and support for gender-equitable norms. Thus, it is also plausible that bundling gender training with the other trainings could shape empowerment impacts. These possibilities inform the focus of our present analysis: comparing the empowerment impacts of agriculture training alone; nutrition training alone; the combination of agriculture and nutrition training; and the combination of agriculture, nutrition, and gender training.

## 3 Study design^1^

### 3.1 Intervention

As described above, ANGeL aimed to assess interventions that can leverage agricultural growth to increase farm household incomes, improve nutrition, and enhance women’s empowerment in Bangladesh. There were three types of interventions:

1) *Agriculture Production:* Facilitating the production of the high-value food commodities that are rich in essential nutrients.
2) *Nutrition Knowledge:* Conducting high-quality behavior change communication (BCC) to improve nutrition knowledge of women and men.
3) *Gender Sensitization:* Undertaking gender sensitization activities that lead to the improvement in the status/empowerment of women and gender parity between women and men.

Accordingly, we implemented a clustered randomized controlled trial with the following arms:^2^

**T-A**: Agricultural Production training

**T-N**: Nutrition Behavior Change Communication (BCC)

**T-AN**: Agricultural Production training and Nutrition BCC

**T-ANG**: Agricultural Production training, Nutrition BCC, and Gender Sensitization

**C**: Control

Training associated with each treatment arm began in July 2016 (after the completion of the baseline survey in January 2016) and ended in December 2017 (before the endline survey which commenced in January 2018); see Table 1. Training took place in the villages where study participants resided, to minimize travel times. Not surprisingly, therefore, approximately 90 percent of all participants reported that training sites were situated within one kilometer from their homes. The lecture-style training – supplemented by directed conversations, practical demonstrations and question-and-answer training formats – was usually held either in meeting rooms or in open courtyards. Each participant received a small stipend (125 Taka) to cover incidental costs associated with attending each training session, or 250 Taka per household per session if both the husband and wife participated. In each treatment village, 25 households took part in these trainings.

**Table 1:** ANGeL training schedule.

Treatment Arm	Training topics	Total sessions	First round (July 24, 2016- March 30, 2017)	2nd round (Refresher training) (April 1, 2017- September 16, 2017)	3rd round (Refresher training) (October 01, 2017- November 30, 2017)	Final review (Key messages) (December 1- December 30, 2017)
T-A	Agriculture	17	6 sessions	6 sessions	4 sessions	1 session
T-N	Nutrition	19	8 sessions	6 sessions	4 sessions	1 session
T-AN	Nutrition + Agriculture	36 (A: 17) (N: 19)	6 sessions (A) + 8 sessions (N)	6 sessions (A) + 6 sessions (N)	4 sessions (A) + 4 sessions (N)	1 session (A) + 1 session (N)
T-ANG	Nutrition + Agriculture + Gender Sensitization	44 (A: 17) (N: 19) (G: 8)	6 sessions (A) + 8 sessions (N)	6 sessions (A) + 6 sessions (N) + 8 sessions (G)*	4 sessions (A) + 4 sessions (N); Key messages on gender integrated with A and N sessions	1 session (A) + 1 session (N)

* The 8 training sessions on gender sensitization (G) were conducted between December 6, 2016 and March 3, 2017.

The agricultural production training (T-A) consisted of 17 sessions held over a 17-month period.^3^ Each session lasted approximately 1.5 h. Topics covered an introduction to the cultivation of high-value crops (fruit and vegetables), using crop calendars to design a year-round system of cultivation, preparation of small plots and homestead gardens, water, pest and fertilizer management, harvest techniques, post-harvest storage, and marketing. Raising poultry and small stock (sheep and goats) was also discussed, with attention to breed selection, feeding, vaccination, and diseases. After the initial training was completed, follow-up sessions included refresher training on key topics, as well as opportunities for participants to discuss their experiences applying the training. Particular care was taken to ensure that both men and women were active participants in these sessions. Training was delivered by sub-assistant agricultural officers (SAAOs) – also referred to as agricultural extension agents (AEAs); these are permanent employees of the Department of Agricultural Extension, which is an agency under the Bangladesh Ministry of Agriculture.

The nutrition treatment arm (T-N) consisted of 19 sessions also held over a 17-month period. Each session lasted approximately 1.5 h. Topics covered included an introduction to the functional roles played by different types of foods, the importance of a balanced diet, micronutrients (vitamin A, iron, iodine, and zinc) and sources of food containing these, age-appropriate complementary foods, optimal breastfeeding practices, maternal nutrition and care, safe food preparation and preservation, hygiene, and handwashing. Sessions included lectures, interactive discussions, games, and cooking demonstrations.^4^ Both husbands and wives were expected to attend each session that was also delivered by AEAs.

Participants in the agricultural production and nutrition (T-AN) treatment arm received the training covered in both the T-N and T-A treatments. Thus, there were 36 training sessions (17 for agriculture and 19 for nutrition), each led by an AEA and lasting approximately 1.5 h.

Agricultural production, nutrition, and gender sensitization (T-ANG) included all material covered in the T-AN treatment arm. For the agriculture and nutrition components, husbands and wives took part in 36 sessions held over a 17-month period with each session lasting approximately 1.5 h. In addition, there were eight sessions on gender sensitization. These sessions, which were based on HKI’s *Nurturing Connections* curriculum (Helen Keller International Bangladesh, 2017) and facilitated by staff hired by HKI, consisted of a set of structured activities that aimed to improve intra-family respect, appreciation, and communication, and improve negotiation skills, to influence women’s empowerment. Recognizing that women’s empowerment is influenced by other household members, this intervention arm also included key household decisionmakers and influencers such as mothers-in-law as participants in the gender sensitization training sessions.

Table 2 provides descriptive statistics on attendance by treatment arm and sex.^5^ Several results are noteworthy. First, there were no treatment arms where the average attendance was lower than 50 percent of all sessions for men or women. Second, women’s attendance was higher than that of men. On average, women attended between 72 and 86 percent of the sessions provided to them compared to 60–80 percent by men. Third, the treatment arm with both agriculture and nutrition (T-AN) had larger numbers of sessions but was generally as well attended (as measured by percentage of sessions attended) as those that had fewer sessions (T-A and T-N). While women attended 86 percent of T-A sessions compared to 84 percent of T-AN sessions, this difference was not significant; women attended a higher percentage of T-AN sessions than T-N sessions (84 percent vs 81 percent), a statistically significant difference (p < 0.05). Men attended 80 percent of T-A sessions vs. 70 percent of T-AN sessions, a statistically significant difference, but their attendance of T-N sessions was not significantly different from attendance of T-AN (71 percent vs. 70 percent). While the mean number of sessions attended by men and women was highest for T-ANG, the percentage of men and women attending 80 percent or more of all sessions was lower than that for other treatment arms. The difference between those attending 80 percent or more of all sessions for T-ANG was significantly lower compared to the other bundled intervention, T-AN, for both women and men. The most frequent reason given by men for missing sessions was that they had to undertake agricultural work in their fields; for women, the most frequent reasons for non-attendance was non-agricultural work and illness.

**Table 2:** Session attendance by treatment arm and sex.

	Means				Test of difference between arms (p-values)				Number of observations
Indicator	T-N	T-A	T-AN	T-ANG	T-N = T-A	T-N = T-AN	T-A = T-AN	T-AN = T-ANG	
Women									
Mean number of sessions attended	15.45	14.84	30.19	31.80	0.02**	0.00***	0.00***	0.00***	2,287
Proportion of sessions attended	0.81	0.86	0.84	0.72	0.00***	0.04**	0.17	0.00***	2,287
Proportion attending 0–20 percent of sessions	0.06	0.04	0.04	0.04	0.14	0.20	0.83	0.85	2,287
Proportion attending 80 percent or more of sessions	0.67	0.77	0.78	0.40	0.00***	0.00***	0.76	0.00***	2,287

Men									
Mean number of sessions attended	13.61	13.75	25.33	26.35	0.67	0.00***	0.00***	0.13	2,096
Proportion of sessions attended	0.71	0.80	0.70	0.60	0.00***	0.58	0.00***	0.00***	2,096
Proportion attending 0–20 percent of sessions	0.09	0.05	0.12	0.12	0.01***	0.15	0.00***	0.93	2,096
Proportion attending 80 percent or more of sessions	0.51	0.65	0.58	0.20	0.00***	0.02**	0.01***	0.00***	2,096

Tests of difference between means by treatment arm: *p < .10; **p < .05; ***p < .01.

Definition of treatment arms: T-N = Nutrition Behavior Change Communication (BCC) training delivered to women and men by agricultural extension agents (AEAs) from the Ministry of Agriculture. T-A = Agricultural Production training delivered to women and men by AEAs. T-AN = Agricultural Production + Nutrition BCC training delivered to women and men by AEAs. T-ANG = Agricultural Production + Nutrition BCC training delivered to women and men by AEAs + gender sensitization activities for women and men conducted by Helen Keller International (HKI).

Most ANGeL participants had a positive experience with their trainers. Nearly all participants (89 percent of males and 90 percent of females) reported that they understood what was taught most of the time or always, and similar percentages described the material presented as “very” or “moderately” informative. It was nearly universal that husbands and wives reported discussing lessons learned from ANGeL trainings together (94 percent of men and 97 percent of women, respectively), with no meaningful difference observed across treatment arms.^6^

### 3.2 Sampling, sample size and survey administration

We aimed to construct a sample size that gave an 80% chance of rejecting the null hypothesis of zero change in outcome indicators at the 0.05 level of significance. For our power calculations, we used parameters derived from the 2011–2012 round of the Bangladesh Integrated Household Survey (BIHS), which is statistically representative of national rural Bangladesh. For outcome indicators, we used per capita daily calorie availability for food security and the household Women’s Empowerment in Agriculture Index score (collected as part of BIHS) for empowerment. These showed that, relative to the control group, 25 clusters and 500 households were needed in each treatment arm to detect a 10% increase in empowerment.

Based on these calculations, we next worked with officials at the Ministry of Agriculture to identify rural upazilas (sub-districts) that were agro-ecologically suitable for agricultural diversification and had good market connectivity. From a list of 484 such upazilas, we collaborated with the Ministry to purposively select 16 upazilas, such that each of the administrative divisions of Bangladesh were represented. Each upazila is divided into “blocks”; each block has an AEA. There were 525 blocks in these 16 upazilas. We randomly selected 10 blocks from each upazila, yielding 160 blocks. These were randomly assigned as follows: 25 blocks to each treatment arm described above; 35 blocks to the control group; and 25 blocks to the second BCC intervention described in footnote 2. We randomly selected one village from each block, then conducted a 100 percent census of households in each of the 160 selected villages. Thereafter, we listed all farm households with at least one child under 24 months from the village census lists. We randomly selected 25 farm households for each of the 160 blocks from village census lists of farm households with at least one child under 24 months, because child nutrition was also an outcome of interest. This yielded 625 households in each treatment arm (2,500 households in total) and 875 households in the control group, for a total sample of 3,375 households.

Baseline data were collected between November 2015 and January 2016. Endline data were collected between January and March 2018. In each household, both the primary female beneficiary and primary male beneficiary were interviewed. Although the male and female beneficiaries were interviewed separately, some modules were answered by only the male (e.g., household demographics, assets and wealth, agricultural production, non-food consumption and expenditures), some were answered by only the female (e.g., food consumption and food security indicators, dietary data, anthropometry, women’s status and decision-making autonomy, experience of IPV), and some were answered separately by each (e.g., data needed to construct the WEAI, gender attitudes, time preferences, agency). Several modules related to empowerment, gender attitudes, IPV, and experience with the program were administered only at endline. In the case of empowerment, the pro-WEAI (see below) was administered at endline but was not available at baseline as it was still under development; instead, at baseline, the abbreviated WEAI (A-WEAI, see Malapit et al. 2017) was fielded. Questions on gender attitudes and IPV were motivated in part by the *Nurturing Connections* curriculum, which was made available after baseline, thus were included only at endline.

## 4 Data, descriptives, and attrition

### 4.1 Outcome variables

Our measure of women’s empowerment at endline is the pro-WEAI, an additive and decomposable index based on the Alkire-Foster methodology adapted from the WEAI (Alkire et al., 2013) for use in agricultural development projects (Malapit, Sraboni, Quisumbing, & Ahmed, 2019). Pro-WEAI is based on a weighted adequacy count across 12 indicators. The 12 indicators attempt to measure three types of agency corresponding to the domains of intrinsic agency, instrumental agency, and collective agency. The indicators of intrinsic agency comprise: autonomy in income, self-efficacy, attitudes about IPV against women, and respect among household members. Instrumental agency indicators include: input in productive decisions, ownership of land and other assets, access to and decisions on financial services, control over use of income, work balance, and visiting important locations. Finally, the collective agency domain includes two indicators: group membership and membership in influential groups. For each of these indicators, individuals are classified as adequate or inadequate based on pre-determined thresholds used in the pro-WEAI. The pro-WEAI is composed of the 3DE sub-index (three domains of empowerment, the pro-WEAI analogue of the five domains of empowerment (5DE) in the WEAI), which measures the extent and depth of empowerment, and the Gender Parity sub-index, which measures gender parity between women and men in the same household. We note that A-WEAI can be computed from the raw data used to compute pro-WEAI, albeit with a different weighting scheme and different adequacy thresholds. Appendix Table A1 provides additional information on the pro-WEAI domains, how they are measured, and how they compare with the A-WEAI domains.

To assess overall empowerment, we use: (1) the individual empowerment score, defined as the weighted sum of the 12 pro-WEAI indicators; this score ranges from 0 to 1; and (2) the individual’s empowerment status, which classifies an individual as empowered if his or her empowerment score is greater than or equal to 75% of the weighted sum of the 12 binary pro-WEAI indicators. Because the empowerment score may not be monotonic in all the 12 indicators, in an appendix, we also analyze impacts on the component indicators; for example, it is possible that ANGeL may increase women’s input into productive decisions but also increase workload. In addition to individual empowerment, we are also interested in gender parity. After calculating an empowerment score for the woman’s partner, we can classify a household as *achieving gender parity* if the woman is empowered, based on the above definition, or if she achieves at least the same empowerment score as her partner; thus, gender parity is a binary indicator at the household level.

Because one of the treatment arms (T-ANG) explicitly aimed to change attitudes towards gender and relationships through its gender sensitization programming, and because men’s and women’s attitudes could also have changed simply through participating in agricultural or nutrition training with their spouses, as described above, a module was added to the endline survey that aimed to capture men’s and women’s degree of agreement with statements related to attitudes. These statements were based on the content of the *Nurturing Connections* curriculum (HKI Bangladesh, 2017). Respondents were asked about their extent of agreement with the statements using a five-point scale, where 1 is “strongly disagree” and 5 is “strongly agree.” Statements were phrased so that they did not always reveal “positive” or more transformative gender attitudes; specifically, these were written so that agreement would be “better” for some statements while disagreement would be “better” for others. Using responses collected separately from women and men, we construct a gender attitudes composite score, which ranges from 9 to 45 and is the sum of the nine statements recoded so that a higher score is associated with more gender-equitable attitudes. In an appendix, we also assess responses to the individual statements. (See Appendix Table A2 for descriptive statistics at endline for the pro-WEAI empowerment indicators, A-WEAI empowerment indicators, and gender attitudes indicators.)

Lastly, it is possible that these new agricultural activities or discussions surrounding gender could lead to unintended consequences. One possibility could be changes in men’s or women’s workloads. There is concern that increasing women’s involvement in agricultural development projects could increase women’s workload in both productive and reproductive work. Nutrition-sensitive agricultural interventions may fail to improve nutritional outcomes if they do not consider time constraints, particularly of rural women who spend a substantial portion of their time in agriculture (Johnston et al., 2018). On the other hand, messaging in the gender sensitization component of ANGeL could have encouraged men to help with household chores that were typically performed by women, leading to a reallocation of workloads. To assess this issue, at endline, we measure women’s and men’s workloads using the 24-h recall module in pro-WEAI.

Another area of concern is that agricultural development projects that attempt to empower women and change gender norms may lead to male backlash, including increases in IPV. It is also possible that ANGeL’s components could have improved spousal relationships or reduced economic insecurity and thus improved emotional well-being of household members, leading to reduced IPV. To measure these effects, at endline, we include questions drawn from the internationally validated standardized IPV modules in the WHO Violence Against Women instrument (Ellsberg & Heise, 2005). The modules ask behaviorally specific questions on a range of abusive acts, a technique shown to maximize disclosure (Ellsberg et al., 2001). Modules were administered to woman respondents, following the WHO protocol on ethical guidelines for conducting research on women’s experiences of IPV (World Health Organization, 2001). Interviewers were adequately trained; women’s informed consent, privacy during interviews, and confidentiality were ensured; only one woman per household was interviewed about experiences with IPV so that other members were not aware this topic was part of the survey; and referrals to a national domestic violence hotline were provided to women disclosing IPV. Female enumerators administered modules on two types of IPV: emotional (4 questions) and physical (6 questions). For each act of violence, women were first asked if their current husband had ever done this. If they reported “yes,” they were asked if it had occurred in the past 6 months and with what frequency (once = 1, a few times = 2, many times = 3). We construct four measures: (1) any emotional violence experienced in the past 6 months; (2) any physical violence experienced in the past 6 months; (3) frequency of emotional violence (0–12, the sum of frequency across all acts, with zero indicating none of these acts occurred) in the past 6 months; and (4) frequency of physical violence (0–18, the sum of frequency across all acts, with zero indicating none of these acts occurred) in the past 6 months. In an appendix, we also assess the occurrence of the individual acts.

### 4.2 Survey characteristics and selection of baseline covariates

To contextualize the study findings, we begin with a summary of characteristics of all households at baseline, as reported in Ahmed et al (2017). Among households that were part of the ANGeL study, the average household size at baseline was 5.5. The dependency ratio – the ratio (expressed as a percentage) of the number of people in the household ages 0–14 and above 60 to the number of working age household members (15–60 years) – was 98.1, slightly higher than the average across all rural Bangladesh, possibly because the ANGeL sample selection criteria required sample households to have at least one child under 24 months. About 65 percent of households owned cultivable land less than half an acre, and just under 80 percent of all households operated <1.5 acres of land. However, farming was by far the most common occupation of the household head across all households (62 percent), followed by business and trade (10 percent). Approximately one-third of the sample only farmed their own land; another third both operated their own land and rented in land; and one third were pure tenants. Sharecropping was widespread.

For inclusion of baseline covariates in our regressions, we include characteristics of the woman, man, and household that are correlated with our outcomes of interest and have few missing values at baseline. These are woman's age (in years); man's age (in years); woman's education (in years); man's education (in years); number of adults in the household; number of children under age 15 years in the household; household's size of operated land (in acres); household wealth index (first component from principal components analysis over household dwelling characteristics and a large set of non-land assets); and dummies for the upazila in which the household lived (binary).

### 4.3 Attrition and baseline descriptives

To develop our estimation sample, we begin with the 3,375 households that comprised the ANGeL sample at baseline. As the calculation of several of our indices requires information from both men and women in the same household, we drop 111 households because they are non-dual-adult households at baseline. This comprises our target baseline sample of 3,264 households. Of this baseline sample, we include 2,739 households in our endline sample. This represents 16.1 percent of the target baseline sample lost to follow up, because: we were not able to collect a full set of outcome data for at least one respondent in the household at endline (423 households); the household migrated (76 households); or the household dropped out of the study, declined to be re-interviewed, or could not be traced (26 households).

Table 3 reports how attrition is correlated with treatment arm and baseline covariates. Coefficients on the treatment arms are small in magnitude. There is no statistically significant impact on attrition of the T-N, T-A or T-ANG treatment arm. Households in the T-AN arm are 2.8 percentage points more likely to attrit than those in the control arm, and this coefficient is weakly significant. However, an F test shows that we cannot reject the null hypothesis that, jointly, attrition does not differ across treatment arms; the p-value for this test is 0.11.

**Table 3:** Correlates of attrition from target baseline sample to estimation sample.

	Coefficients	SE	p-value
T-N	−0.016	0.021	0.444
T-A	0.015	0.025	0.542
T-AN	0.028	0.017	0.089
T-ANG	−0.019	0.020	0.348
Woman's age at baseline (years)	0.000	0.000	0.778
Man's age at baseline (years)	−0.001	0.001	0.304
Woman's education at baseline (years)	0.006	0.002	0.025
Man's education at baseline (years)	−0.007	0.002	0.000
Number of adults in HH at baseline	0.006	0.006	0.333
Number of children under 15 in HH at baseline	−0.001	0.006	0.923
Size of operated land at baseline (acres)	−0.018	0.005	0.001
Wealth index	0.008	0.003	0.013
Constant	0.194	0.042	0.000
			
Upazila fixed effects	YES		
			
Number of observations	3,264		
			
*Test of joint significance*			
F-statistic: Treatment arms	1.913		
p-value	0.105		

*Note:* Dependent variable is coded as 0 if HH is included in sample (Control/T-N/T-A/T-AN/T-ANG only) and 1 if excluded from sample; Standard errors adjusted for clustering at block level. All treatments targeted to men and women.

Definition of treatment arms: T-N = Nutrition Behavior Change Communication (BCC) training delivered to women and men by agricultural extension agents (AEAs) from the Ministry of Agriculture. T-A = Agricultural Production training delivered to women and men by AEAs. T-AN = Agricultural Production + Nutrition BCC training delivered to women and men by AEAs. T-ANG = Agricultural Production + Nutrition BCC training delivered to women and men by AEAs + gender sensitization activities for women and men conducted by Helen Keller International (HKI).

With respect to the baseline covariates we consider, attrition increases very slightly with the woman respondent’s education at baseline and decreases very slightly with the man respondent’s education at baseline. Attrition also decreases slightly for the size of operated land at baseline and increases for the wealth index. Attrition is not significantly associated with other selected baseline covariates and does not differ across upazilas (not shown). While there are some significant relationships between attrition and the baseline covariates, they are small in magnitude.

Table 4 reports the mean values for the baseline covariates selected for inclusion in our regressions. Women in the control group, with a mean age around 26 years, are on average 10 years younger than their husbands; however, they have 6.3 grades of schooling compared to 5.1 for their husbands. Mean land operated at baseline was 1.1 acres for the control group. Magnitudes of baseline covariates are similar across treatment and control arms, although there are small differences. We include baseline covariates in our regressions to help account for these small differences.

**Table 4:** Mean values of baseline covariates, by treatment arm.

	Women					Men				
	Control	T-N	T-A	T-AN	T-ANG	Control	T-N	T-A	T-AN	T-ANG
	Mean	Mean	Mean	Mean	Mean	Mean	Mean	Mean	Mean	Mean
	(SD)	(SD)	(SD)	(SD)	(SD)	(SD)	(SD)	(SD)	(SD)	(SD)
Age (years)	26.56	25.28	26.19	26.58	26.29	36.76	34.75	35.35	36.31	35.72
	(0.32)	(0.25)	(0.36)	(0.36)	(0.29)	(0.68)	(0.77)	(0.67)	(0.93)	(0.65)
Education (years)	6.34	6.55	6.49	5.72	6.38	5.15	5.33	4.97	4.41	5.23
	(0.19)	(0.25)	(0.22)	(0.23)	(0.21)	(0.22)	(0.27)	(0.27)	(0.27)	(0.23)
Number of adults in HH	3.35	3.45	3.30	3.48	3.32					
	(0.10)	(0.14)	(0.15)	(0.15)	(0.12)					
Number of children under 15 in HH	2.17	2.12	2.10	2.31	2.10					
	(0.07)	(0.08)	(0.08)	(0.07)	(0.06)					
Size of operated land at baseline (acres)	1.10	1.24	1.20	1.08	0.96					
	(0.09)	(0.14)	(0.11)	(0.13)	(0.06)					
Wealth index	0.19	−0.09	0.30	−0.01	−0.11					
	(0.20)	(0.37)	(0.26)	(0.24)	(0.28)					
Number of observations	717	520	497	492	513	717	520	497	492	513

*Note:* All treatments targeted to men and women. See notes to Table 3 for definition of treatment arms.

## 5 Empirical specification

Our approach to evaluating ANGeL’s impacts on empowerment, gender attitudes, workload, and IPV takes advantage of the RCT design of the intervention. The randomized assignment of a large sample of eligible households to treatment and control arms helps to reduce the observable and unobservable differences across these arms at baseline. Because our outcomes of interest were collected only at endline, our estimation relies on single-difference estimates. For each outcome of interest, the specification used to assess the single-difference impact of each of the four treatment arms – T-N, T-A, T-AN, T-ANG – relative to the control is described in equation (1):

(1) $$Y_{\mathrm{ib}}=\alpha +\beta_{N}{\mathrm{TN}}_{b}+\beta_{A}{\mathrm{TA}}_{b}+\beta_{\mathrm{AN}}{\mathrm{TAN}}_{b}+\beta_{\mathrm{ANG}}{\mathrm{TANG}}_{b}+\beta_{X}X+\varepsilon_{i}$$

where *Y_ib_* is the outcome of interest for individual *i* residing in block *b*; ${\mathrm{TN}}_{b}$, ${\mathrm{TA}}_{b}$, ${\mathrm{TAN}}_{b}$, and ${\mathrm{TANG}}_{b}$ are dummy variables that take the value of 1 if block *b* was assigned to T-N, T-A, T-AN, and T-ANG, respectively, and takes the value of 0 otherwise; and $\varepsilon_{i}$ is an error term. $\beta_{N}$, $\beta_{A}$, $\beta_{\mathrm{AN}}$, and $\beta_{\mathrm{ANG}}$ represent the single-difference impact estimator for T-N, T-A, T-AN, and T-ANG, respectively. We include a vector of control variables, *X* , for the baseline covariates described above; $\beta_{X}$ represents their associated coefficients. We run separate regressions for outcomes constructed separately for men and women. Standard errors are clustered at the block level, which is the level at which the randomization was conducted.

We estimate ordinary-least-squares regressions for the empowerment score, gender attitudes measures, workload, and frequency of IPV. We estimate probit regressions then calculate marginal effects for binary outcomes: the individual pro-WEAI components, experience of any emotional IPV, and experience of any physical IPV. For each outcome, we conduct Wald tests to assess whether the difference in impacts estimated from various treatment arms are statistically significant. Specifically, we assess whether T-N = T-A; T-N = T-AN; T-A = T-AN; and T-AN = T-ANG. These comparisons allow us to infer how the single interventions compare, depending on whether they focus on agriculture or nutrition; how a combined agriculture and nutrition intervention compares with the single interventions; and how adding gender sensitization to the combined agriculture and nutrition intervention changes impacts. Given variations in attendance across treatment arms, our estimates should be interpreted as intent-to-treat (ITT) estimates.

A common concern when many outcomes are being examined simultaneously is that standard statistical techniques will tend to over-reject the null hypothesis. Although our main outcome variables – such as the empowerment score, whether the woman or man is empowered, and the likelihood of the household achieving gender parity – are well-defined composite measures, concerns about over-rejecting the null hypotheses may be higher when we consider individual component indicators. These include the twelve individual indicators in pro-WEAI, the components of the gender attitudes score, and the types of emotional and physical violence that a woman may report. When we present impacts on these component indicators, we adjust for multiple testing by controlling the false detection rate (FDR) and constructing sharpened q-values following Benjamini et al., 2006, Anderson, 2008. Unadjusted p-values and adjusted q-values are presented in appendix tables for the component indicators.

## 6 Impact estimates

### 6.1 Core results

#### 6.1.1 Pro-WEAI aggregates

Table 5 presents single difference ITT impacts of the ANGeL project on pro-WEAI outcomes: women’s and men’s empowerment scores, whether women and men are empowered, and whether the household achieves gender parity. In the control group at endline, the mean empowerment score for women is 0.59; only 25% of women are empowered, compared to 39% of men, and 47% of control households achieve gender parity. For women’s empowerment outcomes, there are significant positive impacts from all treatment arms (T-A, T-N, T-AN, T-ANG) relative to the control group. The women’s empowerment score increases by 0.04 to 0.07, and the prevalence of empowered women increases by 8 to 13 percentage points. For both the women’s empowerment score and whether women are empowered, the point estimate for impact is highest in the T-ANG arm; however, Wald tests show that the differences in impacts from the added gender sensitization component are not statistically significant. On the other hand, for men’s empowerment outcomes, there are no significant impacts except from the T-N arm which increases men’s empowerment scores by 0.03 and increases the prevalence of empowered men by 10 percentage points. We reject the null hypothesis that T-N = T-A and that T-N = T-AN; the increases in men’s empowerment from the nutrition intervention alone are significantly larger than from the agriculture intervention alone or from a combination of the nutrition and agriculture interventions. Overall, the significant increases in women’s empowerment from each intervention arm occurs without any significant negative impact on men’s empowerment.

**Table 5:** Single-difference impacts on pro-WEAI outcomes.

		Impacts				Test of difference between arms				Number of observations
Indicator	Control	T-N	T-A	T-AN	T-ANG	T-N = T-A	T-N = T-AN	T-A = T-AN	T-AN = T-ANG	
	Mean					p-value				

	(SE)									

Women										
Empowerment score	0.59	0.04***	0.04**	0.04***	0.07***	0.79	0.90	0.90	0.20	2,739
	(0.01)	(0.01)	(0.02)	(0.02)	(0.02)					
Whether empowered	0.25	0.08**	0.07*	0.08**	0.13***	0.81	0.98	0.82	0.28	2,739
	(0.02)	(0.03)	(0.04)	(0.04)	(0.04)					

Men										
Empowerment score	0.67	0.03**	0.01	−0.00	0.00	0.09*	0.01***	0.44	0.68	2,739
	(0.01)	(0.01)	(0.01)	(0.01)	(0.01)					
Whether empowered	0.39	0.10**	0.02	−0.01	0.01	0.07*	0.01**	0.52	0.72	2,739
	(0.03)	(0.04)	(0.04)	(0.04)	(0.04)					

Household										
HH achieves gender parity	0.47	0.05	0.05	0.08*	0.13***	0.95	0.46	0.48	0.28	2,739
	(0.03)	(0.04)	(0.04)	(0.04)	(0.04)					

*Note:* Estimates are intent-to-treat, and estimated as marginal effects for whether empowered and gender parity indicators. The empowerment score is the weighted average of the 12 pro-WEAI indicators. An individual is defined as empowered if s/he reaches the threshold of 75 percent or more of the weighted indicators. A household achieves gender parity if the woman respondent is empowered or her empowerment score is equal to or greater than that of the man respondent in the household. Standard errors adjusted for clustering at block level are in parentheses. *p < .10; **p < .05; ***p < .01. All specifications include as independent variables the treatment indicators, age and education level of both respondents, number of children (0–15 years) in the household, number of adults (15 + years) in the household, size of household operated land, a wealth index and upazila at baseline.

See notes to Table 3 for definition of treatment arms.

The T-AN and T-ANG treatments have positive impacts on gender parity, with a highly significant 13 percentage point increase from the T-ANG arm and a weakly significant 8 percentage point increase from the T-AN arm. Tests of differences across arms show that the impact estimates from T-ANG and T-AN do not significantly differ. Given that a household is classified as achieving gender parity if the woman is either empowered or achieves at least the same empowerment score as the primary man in the household, these results are consistent with the above results for women and men individually.

Because the empowerment score is a weighted average of individual pro-WEAI indicators, not all indicators will necessarily respond in the same way to intervention. Appendix Table A3a, Table A3b, Panel A, presents ANGeL’s impacts on the 12 component indicators of the pro-WEAI for women. Adjustments are shown for multiple testing following Anderson (2008), with q-values controlling the FDR. For women, looking at q-values, we see significant positive impacts from all treatment arms on the indicator for access to/decisions on financial services, in the range of 19 to 23 percentage point increases. No significant impacts are found on other pro-WEAI indicators for women, with multiple testing adjustments.

Appendix Table A3a, Table A3b, Panel B shows impacts on pro-WEAI indicators for men. Based on q-values, we find that the T-N and T-A arms also lead to significant impacts on the access to/decisions on financial services for men, representing 11 and 10 percentage point increases, respectively. No other pro-WEAI indicators for men show significant treatment impacts, with multiple testing adjustments. We discuss possible interpretations of these findings for both women and men in the conclusion.

#### 6.1.2 Gender attitudes composites, women and men

Table 6 shows ANGeL’s impacts on men’s and women’s attitudes regarding gender roles. In the control group at endline, the mean gender attitudes score for both women and men in the control group is 34. In terms of impact, for women’s gender attitudes, only the combined treatments T-AN and T-ANG have significant effects, leading to “improvements” of about 3% and 2%, respectively. For men’s gender attitudes, all treatments that include the nutrition component (T-N, T-AN, T-ANG; but not T-A) significantly improved men’s gender attitudes by 2% to 3%. For both men and women, there do not appear to be statistically significant differences between impacts from the treatment arms.

**Table 6:** Single-difference impacts on gender attitudes score.

		Impacts				Test of difference between arms				Number of observations
Indicator	Control	T-N	T-A	T-AN	T-ANG	T-N = T-A	T-N = T-AN	T-A = T-AN	T-AN = T-ANG	
	Mean					p-value				

	(SE)									

Women										
Total gender attitudes score (9–45)	34.44	0.32	0.27	0.94**	0.80**	0.92	0.20	0.20	0.75	2,739
	(0.28)	(0.45)	(0.49)	(0.43)	(0.40)					

Men										
Total gender attitudes score (9–45)	34.47	0.58*	0.47	0.89***	0.84**	0.76	0.36	0.21	0.89	2,739
	(0.20)	(0.33)	(0.33)	(0.30)	(0.33)					

*Note:* Estimates are intent-to-treat. Standard errors adjusted for clustering at block level are in parentheses. *p < .10; **p < .05; ***p < .01.

See notes to Table 5 for specifications and notes to Table 3 for definition of treatment arms.

To ascertain whether these changes in the gender attitudes score can be attributed to specific questions, we examine the individual items in Appendix Table A4a, Table A4b Panels A and B; estimates for the individual items have been adjusted for multiple hypothesis testing following Anderson (2008). The overall changes in women’s attitudes do not appear to have been driven by any particular item. However, among men, we detect a significant change based on q-values in the item “husbands should help wives with household chores like cooking and taking care of children” only in the T-ANG arm.

#### 6.1.3 Unintended consequences: Workload and IPV

Table 7 assesses the impacts of the ANGeL treatment arms on workloads. In the control group at endline, mean daily workload is just over 9.4 h for both women and men. For women, this is split into about 8 h domestic/care work and about 1.5 h of other productive work; for men, the pattern is reversed, with <1 h of domestic/care work and more than 8.5 h of other productive work. None of the treatment arms has a statistically significantly impact on the workload of women or of men – neither in aggregate, nor by type of work.

**Table 7:** Single-difference impacts on time spent on work.

		Impacts				Test of difference between arms				Number of observations
Indicator	Control	T-N	T-A	T-AN	T-ANG	T-N = T-A	T-N = T-AN	T-A = T-AN	T-AN = T-ANG	
	Mean					p-value				
	(SE)									

Women										
Hours spent on any work	9.49	0.23	0.01	0.06	−0.07	0.45	0.57	0.84	0.61	2,739
	(0.14)	(0.25)	(0.24)	(0.24)	(0.21)					
Hours spent on productive non-domestic work	1.52	−0.02	0.09	−0.12	−0.14	0.57	0.62	0.31	0.93	2,739
	(0.11)	(0.17)	(0.18)	(0.19)	(0.17)					
Hours spent on domestic/care work	7.98	0.25	−0.09	0.19	0.07	0.22	0.82	0.31	0.61	2,739
	(0.15)	(0.23)	(0.24)	(0.22)	(0.19)					

Men										
Hours spent on any work	9.44	0.30	0.34	0.08	0.18	0.91	0.45	0.41	0.75	2,739
	(0.18)	(0.27)	(0.28)	(0.24)	(0.28)					
Hours spent on productive non-domestic work	8.58	0.16	0.16	−0.06	0.08	0.98	0.48	0.46	0.63	2,739
	(0.19)	(0.30)	(0.27)	(0.25)	(0.27)					
Hours spent on domestic/care work	0.86	0.14	0.18	0.15	0.10	0.77	0.95	0.82	0.75	2,739
	(0.09)	(0.15)	(0.14)	(0.14)	(0.13)					

*Note:* Estimates are intent-to-treat. Standard errors adjusted for clustering at block level are in parentheses. *p < .10; **p < .05; ***p < .01.

Domestic/care work includes time spent on shopping/getting services, weaving/sewing/textiles, cooking, domestic work, caring for children, and caring for adults.

See notes to Table 5 for specifications and notes to Table 3 for definition of treatment arms.

Table 8 presents ANGeL’s impacts on IPV. In the control group at endline, over the preceding six months, 21% of women report experiencing any emotional violence, and 7% report experiencing any physical violence. Impact estimates show that, relative to the control group, none of the treatment arms significantly changes the prevalence of women reporting any emotional violence in the preceding six months nor the prevalence reporting any physical violence in the preceding six months, relative to the control group. However, Wald tests suggest that T-ANG leads to a weakly significant increase in prevalence of emotional IPV relative to T-AN (point estimate of 0.07 relative to −0.02) as well as a weakly significant increase in prevalence of physical IPV relative to T-AN (point estimate of 0.02 relative to −0.02). Other differences between treatment arms in impacts on IPV prevalence are not statistically significant. In terms of reported frequency of IPV, the gender sensitization arm (T-ANG) leads to a weakly significant increase in the reported frequency both of emotional violence (a small point estimate of 0.32 on a scale from 0 to 12, but about a 50% increase relative to control) and of physical violence (a small point estimate of 0.15 on a scale from 0 to 18, but a 100% increase relative to control). Wald tests suggest that T-ANG leads to weakly significant increases in frequency of emotional IPV relative to T-AN, although no other differences between treatment arms in impacts on IPV frequency are statistically significant.

**Table 8:** Single-difference impacts on IPV indicators, last six months (Women).

		Impacts				Test of difference between arms				Number of observations
Indicator	Control	T-N	T-A	T-AN	T-ANG	T-N = T-A	T-N = T-AN	T-A = T-AN	T-AN = T-ANG	
	Mean					p-value				
	(SE)									

*In last six months*										
Emotional violence	0.21	−0.01	0.02	−0.02	0.07	0.48	0.99	0.49	0.10*	2,739
	(0.03)	(0.04)	(0.05)	(0.05)	(0.04)					
Physical violence	0.07	−0.01	0.02	−0.02	0.02	0.14	0.77	0.13	0.09*	2,732
	(0.01)	(0.02)	(0.02)	(0.02)	(0.02)					
Frequency of emotional violence (0–12)	0.64	−0.04	0.01	−0.03	0.32*	0.77	0.96	0.82	0.09*	2,739
	(0.11)	(0.15)	(0.17)	(0.17)	(0.19)					
Frequency of physical violence (0–18)	0.15	0.03	0.05	−0.00	0.15*	0.78	0.68	0.49	0.13	2,739
	(0.04)	(0.06)	(0.05)	(0.07)	(0.08)					

*Note:* Estimates are intent-to-treat, and estimated as marginal effects for ever experience of violence indicators. Standard errors adjusted for clustering at block level are in parentheses. *p < .10; **p < .05; ***p < .01. Emotional violence includes insulted you or made you feel bad about yourself; belittled or humiliated you in front of other people; did things to scare or intimidate you on purpose; threatened to hurt you or someone you care about. Physical violence includes slapped your or threw something at you that could hurt you; pushed you or shoved you or pulled your hair; hit you with his fist or with something else that could hurt you; kicked you, dragged you or beat you up; choked or burnt you on purpose; threatened to use or actually used a gun, knife or other weapon against you.

See notes to Table 5 for specifications and notes to Table 3 for definition of treatment arms.

We take a closer look at the prevalence and frequency of reporting the individual acts of emotional and physical IPV in Appendix Table A5a, Table A5b, Table A6a, Table A6b. The point estimates for prevalence or frequency of each individual act in the T-ANG arm are consistently slightly larger or the same relative to the other treatment arms; for some individual acts, unadjusted p-values suggest T-ANG leads to (weakly) significant increases in prevalence and frequency (for example, for “done things to scare or intimidate you on purpose,” “threatened to hurt you or someone you care about,” and “kicked you, dragged you, or beat you up”). However, after adjusting for multiple hypothesis testing, we find no statistically significant impacts of any of the treatment arms relative to the control arm, in either the reported prevalence or reported frequency of individual acts of IPV.

Taken together, results show few significant impacts on IPV; but T-ANG shows isolated weakly significant increases in prevalence or frequency of IPV, with small point estimates, relative to the control or to T-ANG. Drawing conclusions about these impacts is challenging, both because they are isolated and weakly significant, and because their interpretation is ambiguous. In particular, it is unclear whether they might imply that adding a gender sensitization component leads women to experience more frequent acts of IPV, or alternatively leads women to be more sensitized to IPV and thus more likely to recall and report instances to the enumerator (given IPV is sensitive and typically underreported). While we cannot definitively distinguish between these explanations, we note that results on women’s and men’s attitudes toward IPV in Appendix Table A3a, Table A3b are suggestive. In Panel A, q-values suggest no significant impacts on women’s attitudes from any treatment; point estimates are positive and larger from T-ANG than the other arms (p-value = 0.09, without multiple testing adjustments), suggesting at least the possibility of T-ANG making women less accepting of violence, though this is not conclusive. In Panel B, q-values also show no significant impacts on men’s attitudes from any treatment; although again not conclusive, all point estimates are positive, thus there is at least no evidence that T-ANG increased men’s perceptions of acceptability of violence. Exploring this further, we assess impacts on women’s and men’s self-reported acceptance of IPV under each individual scenario asked about, in Appendix Table A6a, Table A6b Panels A and B. Specifically, we assess the proportion of respondents who answered “No” regarding whether a husband was justified in hitting or beating his wife in the following situations: if she goes out without telling him; if she neglects the children; if she argues with him; if she refuses to have sex with him; if she burns the food. Again, based on q-values, we find no significant decrease in the proportion of men or women reporting that IPV is not justified (i.e., no significant increase in viewing IPV as justified). Nor do we see a stark discrepancy in treatment impacts on women’s and men’s attitudes (e.g., if women’s acceptance of IPV drastically reduced due to T-ANG, while men’s stayed the same, there could be potential backlash; we cannot rule this out, but we do not see clear evidence). Thus, we view the results on IPV as inconclusive. Because noting potential risks of interventions is important, we nonetheless recommend monitoring for changes in IPV when adding a gender sensitization component to agriculture and nutrition trainings.

### 6.2 Sensitivity to choice of empowerment indicator and estimation strategy

ANGeL has significant impacts on the empowerment score as measured by pro-WEAI, the probability that a woman is empowered, and the likelihood that the household attains gender parity. As pro-WEAI is a relatively new index in the WEAI-based family of indicators, it is useful to compare it with another WEAI variant that is widely used in population-based surveys, the Abbreviated-WEAI (A-WEAI) (Malapit et al. 2017). In the instance of ANGeL, where we can construct A-WEAI at both baseline and endline, analyzing impacts on A-WEAI also allows us to explore whether single-difference estimation in this study shows qualitatively different results than double-difference.

**Fig. 1:**
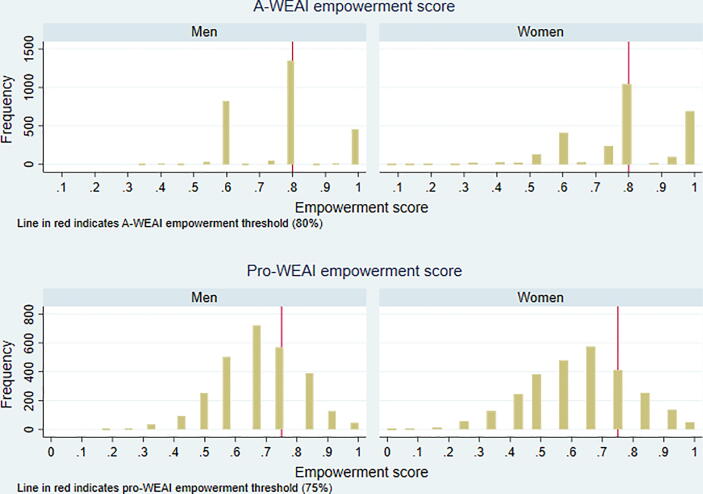
Empowerment score distributions for pro-WEAI and A-WEAI, by sex, at endline. Notes: Empowerment score is the weighted sum of the pro-WEAI/A-WEAI indicators. See Appendix Table 1 for definitions of pro-WEAI and A-WEAI indicators.

To address the former point, we estimate single-difference impacts on A-WEAI outcomes for both women and men, in Table 9. Unlike pro-WEAI, we are only able to detect a weakly significant impact of the T-ANG treatment on the women’s empowerment score measured using A-WEAI, and no other significant impacts. To understand why these results differ, it is useful to note that pro-WEAI has 12 indicators, compared to A-WEAI’s six, and that it was designed to capture more aspects of empowerment that are relevant to agricultural development projects. Moreover, the cut-offs for the individual indicators in pro-WEAI are stricter, because pro-WEAI was designed for projects that have explicit empowerment objectives. Having more indicators introduces more variability in the aggregate index, making it more sensitive. This is particularly relevant if many individuals are clustering or bunching around the threshold for adequacy. Fig. 1, which shows the distributions of A-WEAI and pro-WEAI for men and women, is revealing. Although one would need to be adequate in 80% of the indicators to be classified as empowered in A-WEAI compared to 75% in pro-WEAI, the distribution of the pro-WEAI and A-WEAI scores is quite different. The extent of bunching around the threshold value is much higher with A-WEAI, making it difficult to measure improvements in empowerment.

**Table 9:** Single-difference impacts on A-WEAI outcomes.

		Impacts				Test of difference between arms				Number of observations
Indicator	Control	T-N	T-A	T-AN	T-ANG	T-N = T-A	T-N = T-AN	T-A = T-AN	T-AN = T-ANG	
	Mean					p-value				
	(SE)									

Women										
A-WEAI: Empowerment score	0.79	0.01	−0.00	−0.00	0.02*	0.49	0.51	0.91	0.06*	2,739
	(0.01)	(0.02)	(0.02)	(0.01)	(0.01)					
A-WEAI: Whether empowered	0.67	−0.01	−0.01	0.02	0.04	0.96	0.52	0.50	0.49	2,732
	(0.02)	(0.04)	(0.04)	(0.04)	(0.03)					

Men										
A-WEAI: Empowerment score	0.76	0.01	−0.00	0.01	0.02	0.53	0.81	0.42	0.68	2,739
	(0.01)	(0.01)	(0.01)	(0.01)	(0.01)					
A-WEAI: Whether empowered	0.65	0.02	0.00	0.04	0.04	0.75	0.63	0.44	0.96	2,739
	(0.03)	(0.04)	(0.04)	(0.04)	(0.04)					

Household										
A-WEAI: HH achieves gender parity	0.75	−0.02	−0.00	0.00	0.02	0.62	0.53	0.87	0.67	2,732
	(0.02)	(0.03)	(0.03)	(0.03)	(0.03)					

*Note:* Estimates are intent-to-treat, and estimated as marginal effects for whether empowered and gender parity indicators. The empowerment score is the weighted average of the six A-WEAI indicators. An individual is defined as empowered if s/he reaches the threshold of 80 percent or more of the weighted indicators. A household achieves gender parity if the woman respondent is empowered or her empowerment score is equal to or greater than that of the man respondent in the household. Standard errors adjusted for clustering at block level are in parentheses. *p < .10; **p < .05; ***p < .01.

See notes to Table 5 for specifications and notes to Table 3 for definition of treatment arms.

To address the latter point, we also estimate double-difference impacts of the ANGeL treatment arms on A-WEAI, in Appendix Table A7. We find very similar results to the single-difference results in Table 9, suggesting the estimation strategy does not drive empowerment results at least in the case of A-WEAI.

## 7 Discussion and conclusion

ANGeL increased both women’s and men’s empowerment, raised the prevalence of households achieving gender parity, and led to small improvements in the gender attitudes of both women and men. Specifically, we find significant increases in women’s empowerment scores and empowerment status from all treatment arms – T-N, T-A, T-AN, T-ANG – but with no significant differences across these. Relative to the 25% of women in the control group that are empowered, the magnitudes of the impacts on women’s empowerment status – increases between 8 and 13 percentage points – are meaningful. Further, the positive impacts of these treatment arms on women did not coincide with reductions in men’s empowerment. No treatment led to significant negative effects on men’s empowerment, and one, T-N, increased both men’s empowerment scores and empowerment status. Both T-AN and T-ANG led to increases in households achieving gender parity. T-AN and T-ANG had small, positive impacts (significant or weakly significant) on women’s gender attitudes with no significant differences between these. For men, we find significant or weakly significant improvements in gender attitudes from T-N, T-AN and T-ANG, with no significant differences across any of these. We find no evidence of unintended impacts on workloads but find inconclusive evidence about impacts on IPV.

Several points are worth noting in our findings. First, patterns of empowerment impacts differ between women and men. For women, *all* bundled and single interventions improve empowerment. The bundled intervention with a gender sensitization component (T-ANG) leads to slightly larger point estimates on women’s empowerment score and status, but these are not significantly different from impacts of the T-AN intervention. For men by contrast, *only* the intervention with a nutrition component alone increases empowerment score and status.

We cannot answer conclusively why patterns of impact differ between women and men. We note, however, that empowerment impacts for both women and men appear to be driven by increases in access to/decisions regarding credit. Recall that this indicator requires meeting at least one of the following conditions: (1) belonging to a household that used a source of credit in the past year *and* participating in at least one sole or joint decision about it, (2) belonging to a household that did not use credit in the past year but could have if it wanted to from at least one source, or (3) having access, solely or jointly, to a financial account. Given that the ANGeL interventions did not directly involve credit, one possible interpretation of our findings is that trainings in a particular domain increased decision-making on use of credit for that domain. For example, if women previously had relatively little input into decision-making around the use of credit in agriculture, their participation in the agricultural treatment arm may have led to their greater involvement in decisions about using credit for agricultural activities. Men may have had high levels of participation in decision-making around use of credit for agriculture, but relatively lower levels of participation in decision-making around use of credit for food and nutrition. In essence, women and men both may have become more involved in areas of decisionmaking around credit that they previously were not, if trained together in that area. An implication could be that, if men were previously less involved in nutrition, the T-N training may have been particularly empowering for men; any arm that bundled agricultural training and thus also increased women’s involvement in a domain where women were previously less involved, may have offset the effect for men. If so, this could be a potential explanation for why bundling agriculture with nutrition was less empowering for men than nutrition alone, while the same pattern did not appear for women.

Second, and related to the point above, the design of ANGeL does not allow us to assess the extent to which including men and women within the same treatment arms contributed to our results, but plausibly it played a role. In particular, the positive impacts of all treatment arms on women’s empowerment outcomes—and the absence of a detectable difference across arms—may have arisen from all implementation modalities providing information to both husbands and wives when they were together. Qualitative work conducted as part of the impact evaluation supports this possibility; both men and women beneficiaries in the T-ANG arm indicate that joint training has facilitated joint decisionmaking.

“After training, taking a joint decision is easier. It’s better than before. Earlier, I used to buy things for cooking. I used to cook as I wish. Now, we take decision together and we can avoid any confusion or complexities.” –T-ANG woman beneficiary

“As we both attended ANGeL training, we both know that taking decisions together is good. After participating in ANGeL, my wife could take the right decision, and sometimes the husband’s decision is wrong.” –T-ANG man beneficiary

The careful implementation of the agricultural treatment arm – ensuring active inclusion of both men and women – may have led to women having more of a voice in decisionmaking. Engaging men with interventions on nutrition may have been impactful because it exposed them to messages that they may not receive in traditional nutrition BCC, which often targets only women, or because it engaged men in an area where women typically hold responsibilities. The role of engaging men and women jointly in interventions, and how best to do so, is a promising area for future research.

Third, for women, impacts on the composite measure of gender attitudes do not appear to be driven by agreement with any particular statement. For men, however, positive impacts on the composite appear to be driven by greater agreement with the statement, “Husbands should help wives with household chores like cooking and taking care of children.” Among men, treatments that include a nutrition component also lead to significantly higher proportions agreeing with this statement; for example, Wald tests (not shown) indicate that the impact of T-A is significantly smaller than impacts of T-AN. This suggests that men’s participation with their wives in the nutrition component – even without explicit gender sensitization – may lead them to see greater importance in women’s typical roles around child nutrition. Nutrition trainings that emphasize the importance of child nutrition may valorize for men some of women’s responsibilities that were previously not recognized. Given that changes in attitudes are important for longer-term gender-transformative effects, these results may be meaningful. That said, we see no reductions in women’s actual reported workloads based on 24-hour recall, thus these effects remain to be demonstrated. In any case we may not expect changes in attitudes to translate to immediate action. The possibility of gender-transformative effects, as well as the persistence of changes in attitudes beyond the intervention, are additional promising areas for future research.

Fourth, we do not see evidence of unintended harm to women in terms of workloads. However, in terms of IPV, the evidence is inconclusive. Results show few significant impacts overall on IPV, but isolated weakly significant increases in prevalence or frequency from T-ANG with small point estimates, relative to the control or to T-AN. Although it is hard to know how meaningful these isolated weakly significant impacts are, we highlight them given the importance of potential risks. On one hand, results could suggest that addition of the gender sensitization component slightly increased the frequency of women experiencing emotional and physical IPV, which is concerning and could reflect male backlash; we do not find evidence for this possibility but cannot rule it out. On the other hand, results could reflect that the addition of the gender sensitization component increased women’s awareness of what constitutes violence (particularly emotional IPV, which is typically underreported and which may have been previously perceived as acceptable) and made women more likely to recall and *report* certain instances to the enumerator. Although our findings on IPV are not straightforward to interpret, they highlight the importance of monitoring unintended harm to women when implementing gender-transformative interventions and suggest the need for further research.

Taken together, our results suggest some potential benefits of bundling nutrition and gender components with an agricultural development intervention, although effects appear to differ by gender and are not consistently large. In the case of men, benefits seem to arise from participation in the nutrition training itself, rather than specifically from the nutrition training being bundled with other components. For women, benefits appear larger in some cases from bundled interventions (e.g., T-ANG vs. T-AN; or T-AN vs. T-A or T-N), but not in all cases, and differences are rarely statistically significant. For example, the point estimates for impacts on women’s empowerment and households achieving gender parity are higher for T-ANG than T-AN; but the differences are not statistically significant.

An unanswered question that arises from our study relates to the ideal number of training sessions included in these treatment arms. While the proportion of individuals with very low attendance (0–20 percent) tended to be similar across arms, for both women and men, the arm that required attendance at the largest number of sessions (T-ANG) had the lowest proportion of sessions attended by both men and women. It would be valuable to undertake an ANGeL-type RCT, but randomly vary the intensity of training to see if similar impacts could be achieved with fewer training sessions, thus lowering the cost of the intervention. A second unanswered question relates to the fact that women and men attended these sessions together. It is possible that creating a neutral, non-threatening space where couples could discuss and problem solve on topics important to their households was the key factor that generated these impacts; an RCT which retained the same type of training but randomly assigned treatment to men-only, women-only and men and women together would shed light on this. Future studies with similar additive designs would be useful to build the evidence on benefits of bundling interventions, particularly with costing analysis that allows estimating the cost-effectiveness of such strategies and informing scale-up.

## CRediT authorship contribution statement

**Agnes Quisumbing:** Conceptualization, Writing - original draft, Writing - review & editing, Methodology, Funding acquisition. **Akhter Ahmed:** Conceptualization, Investigation, Supervision, Project administration, Funding acquisition, Writing - original draft, Writing - review & editing. **John Hoddinott:** Conceptualization, Writing - original draft, Writing - review & editing, Methodology. **Audrey Pereira:** Software, Formal analysis, Writing - original draft, Writing - review & editing. **Shalini Roy:** Conceptualization, Formal analysis, Writing - original draft, Writing - review & editing, Methodology.

## Declaration of Competing Interest

The authors declare that they have no known competing financial interests or personal relationships that could have appeared to influence the work reported in this paper.

## Figures and Tables

**Table A1:** Comparing Pro-WEAI and A-WEAI domains and indicators.

Pro-WEAI domain	Pro-WEAI indicator name	Pro-WEAI definition	A-WEAI domain	A-WEAI indicator name	A-WEAI definition
Intrinsic agency	Autonomy in income	More motivated by own values than by coercion or fear of others’ disapproval *Weight = 1/12*			
Intrinsic agency	Self-efficacy	“Agree” or greater on average with self-efficacy questions: *New General Self-Efficacy Scale*^A^ score>=32 *Weight = 1/12*			
Intrinsic agency	Attitudes about intimate partner violence against women	Believes husband is NOT justified in hitting or beating his wife in all 5 scenarios: ^B^ 1) She goes out without telling him 2) She neglects the children 3) She argues with him 4) She refuses to have sex with him 5) She burns the food *Weight = 1/12*			
Intrinsic agency	Respect among household members	Meets ALL of the following conditions related to their spouse, the other respondent, or another household member: 1) Respondent respects relation (MOST of the time) AND 2) Relation respects respondent (MOST of the time) AND 3) Respondent trusts relation (MOST of the time) AND 4) Respondent is comfortable disagreeing with relation (MOST of the time) *Weight = 1/12*			
Instrumental Agency	Input in productive decisions	Meets at least ONE of the following conditions for ALL of the agricultural activities they participate in 1) Makes related decision solely, 2) Makes the decision jointly and has at least some input into the decisions 3) Feels could make decision if wanted to (to at least a MEDIUM extent) *Weight = 1/12*	Production	Input in productive decisions	Adequate if individual participates in and makes decisions, has input in decisions, or feels she could make decisions (if desired) about at one agricultural activity *Weight = 1/5*
Instrumental Agency	Ownership of land and other assets	Owns, either solely or jointly, at least ONE of the following: (updated March 2020) 1) Any three assets 2) Land *Weight = 1/12*	Resources	Ownership of assets	Adequate if individual owns at least one major asset or at least two minor assets *Weight = 2/15*
Instrumental Agency	Access to and decisions on financial services	Meets at least ONE of the following conditions: 1) Belongs to a household that used a source of credit in the past year AND participated in at least ONE sole or joint decision about it 2) Belongs to a household that did not use credit in the past year but could have if wanted to from at least ONE source 3) Has access, solely or jointly, to a financial account *Weight = 1/12*	Resources	Access to and decisions about credit	Adequate if individual makes decisions about at least one source of credit accessed by her/his household *Weight = 1/15*
Instrumental Agency	Control over use of income	Has input in decisions related to how to use BOTH income and output from ALL of the agricultural activities they participate in AND has input in decisions related to income from ALL non-agricultural activities they participate in, unless no decision was made *Weight = 1/12*	Income	Control over use of income	Adequate if individual participates in and has input in decisions about income generated from an activity or she/he makes decisions, has input in decisions, or feels she/he could make decisions (if desired) about employment or major household expenditures *Weight = 1/5*
Instrumental Agency	Work balance	Works<10.5 h per day: Workload = time spent in primary activity + (1/2) time spent in childcare as a secondary activity *Weight = 1/12*	Time	Workload	Adequate if individual worked<10.5 h during the previous day *Weight = 1/5*
Instrumental Agency	Visiting important locations	Meets at least ONE of the following conditions: 1) Visits at least TWO locations at least ONCE PER WEEK of [city, market, family/relative], or 2) Visits least ONE location at least ONCE PER MONTH of [health facility, public meeting] *Weight = 1/12*			
Collective Agency	Group membership	Active member of at least ONE group *Weight = 1/12*	Leadership	Group member	Adequate if individual is an active member of at least one group *Weight = 1/5*
Collective Agency	Membership in influential groups	Active member of at least ONE group that can influence the community to at least a MEDIUM extent *Weight = 1/12*			

Source: Malapit et al., 2019, Malapit et al., 2017.

^A^ The New General Self-efficacy Scale (NGSE) is a validated scale to measure self-efficacy, or a person’s capabilities and ability to reach their goals (Chen, Gully, & Eden, 2001). The questions can be found in Appendix C, module G8(B).

^B^ These scenarios are based on previously validated items from the Demographic and Health Surveys.

**Table A2:** Empowerment diagnostics of men and women by treatment arm, endline.

	Women					Men				
	Control	T-N	T-A	T-AN	T-ANG	Control	T-N	T-A	T-AN	T-ANG
	Mean	Mean	Mean	Mean	Mean	Mean	Mean	Mean	Mean	Mean
	(SD)	(SD)	(SD)	(SD)	(SD)	(SD)	(SD)	(SD)	(SD)	(SD)
*Pro-WEAI indicators*										
Autonomy in income	0.76	0.83	0.77	0.75	0.83	0.95	0.96	0.96	0.91	0.93
	(0.03)	(0.02)	(0.04)	(0.04)	(0.03)	(0.01)	(0.01)	(0.01)	(0.02)	(0.02)
Self-efficacy	0.50	0.54	0.57	0.54	0.54	0.60	0.66	0.64	0.61	0.54
	(0.04)	(0.04)	(0.04)	(0.05)	(0.04)	(0.04)	(0.04)	(0.04)	(0.04)	(0.04)
Attitudes about intimate partner violence against women	0.62	0.64	0.67	0.64	0.71	0.70	0.78	0.71	0.72	0.73
	(0.04)	(0.03)	(0.04)	(0.04)	(0.04)	(0.03)	(0.03)	(0.03)	(0.03)	(0.03)
Respect among household members	0.71	0.70	0.72	0.75	0.72	0.82	0.81	0.81	0.78	0.76
	(0.04)	(0.03)	(0.04)	(0.04)	(0.03)	(0.03)	(0.03)	(0.04)	(0.04)	(0.04)
Input in productive decisions	0.86	0.91	0.89	0.89	0.89	0.90	0.90	0.89	0.87	0.91
	(0.02)	(0.02)	(0.02)	(0.02)	(0.02)	(0.02)	(0.02)	(0.03)	(0.02)	(0.02)
Ownership of land and other assets	0.81	0.84	0.80	0.83	0.88	0.99	1.00	0.99	0.99	0.99
	(0.03)	(0.03)	(0.04)	(0.03)	(0.02)	(0.00)	(0.00)	(0.01)	(0.01)	(0.00)
Access to and decisions on financial services	0.62	0.86	0.89	0.88	0.86	0.78	0.89	0.88	0.81	0.86
	(0.02)	(0.03)	(0.02)	(0.02)	(0.02)	(0.02)	(0.02)	(0.02)	(0.02)	(0.02)
Control over use of income	0.80	0.86	0.83	0.83	0.87	0.86	0.85	0.84	0.83	0.87
	(0.03)	(0.02)	(0.02)	(0.02)	(0.03)	(0.02)	(0.03)	(0.03)	(0.03)	(0.03)
Work balance	0.48	0.46	0.48	0.46	0.50	0.53	0.47	0.48	0.54	0.53
	(0.03)	(0.04)	(0.04)	(0.05)	(0.05)	(0.02)	(0.03)	(0.03)	(0.04)	(0.04)
Visiting important locations	0.24	0.25	0.32	0.28	0.32	0.49	0.58	0.53	0.52	0.51
	(0.03)	(0.04)	(0.04)	(0.04)	(0.05)	(0.03)	(0.04)	(0.04)	(0.04)	(0.05)
Group membership	0.45	0.47	0.45	0.47	0.54	0.29	0.35	0.28	0.30	0.35
	(0.03)	(0.04)	(0.05)	(0.03)	(0.04)	(0.03)	(0.04)	(0.04)	(0.04)	(0.04)
Membership in influential groups	0.21	0.23	0.19	0.24	0.25	0.17	0.20	0.16	0.14	0.15
	(0.02)	(0.03)	(0.04)	(0.03)	(0.04)	(0.02)	(0.02)	(0.03)	(0.02)	(0.02)
*Gender attitudes score*										
Total gender attitudes score (9–45)	34.44	34.80	34.76	35.31	35.20	34.47	35.01	34.89	35.26	35.28
	(0.28)	(0.38)	(0.44)	(0.34)	(0.30)	(0.20)	(0.27)	(0.28)	(0.22)	(0.24)
*Gender attitudes statements*										
I make important contributions to my family	4.68	4.68	4.68	4.70	4.63	4.60	4.68	4.72	4.66	4.66
	(0.03)	(0.05)	(0.05)	(0.04)	(0.05)	(0.04)	(0.03)	(0.04)	(0.05)	(0.05)
Some adults in my family do not make important contributions to the family	2.40	2.39	2.40	2.29	2.37	2.46	2.40	2.32	2.24	2.37
	(0.09)	(0.15)	(0.14)	(0.13)	(0.10)	(0.09)	(0.11)	(0.08)	(0.10)	(0.11)
I make important contributions to my community	3.23	3.19	3.39	3.42	3.42	3.77	3.88	3.79	3.87	3.68
	(0.07)	(0.10)	(0.09)	(0.10)	(0.10)	(0.06)	(0.07)	(0.07)	(0.07)	(0.08)
I sometimes refrain from voicing my opinion because I fear being ignored/ridiculed	3.46	3.43	3.38	3.29	3.27	3.19	3.12	3.01	3.05	2.91
	(0.08)	(0.08)	(0.11)	(0.11)	(0.11)	(0.08)	(0.11)	(0.07)	(0.11)	(0.10)
I have a hard time saying positive things about myself	3.51	3.50	3.49	3.45	3.47	3.36	3.33	3.26	3.35	3.21
	(0.07)	(0.08)	(0.11)	(0.11)	(0.07)	(0.08)	(0.11)	(0.08)	(0.10)	(0.10)
Women should stand up for themselves to get what they want	4.67	4.75	4.70	4.74	4.72	4.45	4.42	4.35	4.46	4.49
	(0.03)	(0.04)	(0.05)	(0.03)	(0.03)	(0.05)	(0.05)	(0.07)	(0.05)	(0.05)
Women are usually very busy with work that benefits the household	4.74	4.73	4.76	4.75	4.77	4.51	4.54	4.46	4.61	4.62
	(0.03)	(0.05)	(0.03)	(0.03)	(0.03)	(0.04)	(0.04)	(0.05)	(0.03)	(0.04)
Husbands should help wives with household chores like cooking and taking care of children	4.62	4.68	4.64	4.70	4.67	4.42	4.53	4.45	4.53	4.59
	(0.04)	(0.06)	(0.04)	(0.04)	(0.04)	(0.04)	(0.04)	(0.05)	(0.04)	(0.04)
We can change culture/tradition regarding what men/women do and how they relate to each other	3.87	4.10	3.86	4.04	4.11	3.74	3.82	3.70	3.78	3.75
	(0.10)	(0.08)	(0.09)	(0.11)	(0.09)	(0.07)	(0.08)	(0.09)	(0.07)	(0.07)
*A-WEAI outcomes*										
A-WEAI: Empowerment score	0.79	0.79	0.79	0.78	0.82	0.76	0.77	0.76	0.77	0.78
	(0.01)	(0.02)	(0.02)	(0.01)	(0.01)	(0.01)	(0.01)	(0.01)	(0.01)	(0.01)
A-WEAI: Whether empowered	0.67	0.64	0.65	0.68	0.73	0.65	0.66	0.64	0.67	0.69
	(0.02)	(0.04)	(0.04)	(0.04)	(0.03)	(0.03)	(0.04)	(0.04)	(0.04)	(0.03)
A-WEAI: HH achieves gender parity	0.75	0.73	0.76	0.76	0.79	0.75	0.73	0.76	0.76	0.79
	(0.02)	(0.03)	(0.03)	(0.03)	(0.03)	(0.02)	(0.03)	(0.03)	(0.03)	(0.03)
A-WEAI: Input in productive decisions	0.97	0.98	0.98	0.98	0.97	0.99	1.00	0.99	0.99	0.99
	(0.01)	(0.01)	(0.01)	(0.01)	(0.01)	(0.00)	(0.00)	(0.00)	(0.01)	(0.00)
A-WEAI: Ownership of assets	0.96	0.97	0.95	0.97	0.97	1.00	1.00	0.99	1.00	1.00
	(0.01)	(0.01)	(0.02)	(0.01)	(0.01)	(0.00)		(0.00)	(0.00)	(0.00)
A-WEAI: Access to and decisions about credit	0.81	0.78	0.82	0.81	0.83	0.96	0.96	0.97	0.96	0.97
	(0.02)	(0.03)	(0.02)	(0.03)	(0.02)	(0.01)	(0.01)	(0.01)	(0.01)	(0.01)
A-WEAI: Control over use of income	0.94	0.97	0.96	0.96	0.96	1.00	1.00	1.00	1.00	1.00
	(0.01)	(0.01)	(0.01)	(0.02)	(0.01)	(0.00)	(0.00)		(0.00)	(0.00)
A-WEAI: Group member	0.45	0.47	0.45	0.47	0.54	0.29	0.35	0.28	0.30	0.35
	(0.03)	(0.04)	(0.05)	(0.03)	(0.04)	(0.03)	(0.04)	(0.04)	(0.04)	(0.04)
A-WEAI: Workload	0.67	0.62	0.64	0.61	0.68	0.56	0.50	0.52	0.58	0.57
	(0.03)	(0.05)	(0.04)	(0.05)	(0.04)	(0.02)	(0.03)	(0.03)	(0.04)	(0.04)
Number of observations	717	520	497	492	513	717	520	497	492	513

*Note:* The A-WEAI empowerment score is the weighted average of the 6 A-WEAI indicators. An individual is defined as empowered if s/he reaches the threshold of 80 percent or more of the weighted indicators. A household achieves gender parity if the woman respondent is empowered or her empowerment score is equal to or greater than that of the man respondent in the household.

Treatment arms: T-N = Nutrition Behavior Change Communication (BCC) training delivered to women and men by agricultural extension agents (AEAs) from the Ministry of Agriculture. T-A = Agricultural Production training delivered to women and men by AEAs. T-AN = Agricultural Production + Nutrition BCC training delivered to women and men by AEAs. T-ANG = Agricultural Production + Nutrition BCC training delivered to women and men by AEAs + gender sensitization activities for women and men conducted by Helen Keller International (HKI).

**Table A3a:** (Panel A): Single-difference impacts on pro-WEAI outcomes (Women).

		Impacts				Number of observations
Indicator	Control	T-N	T-A	T-AN	T-ANG	
	Mean	Coeff	Coeff	Coeff	Coeff	
	(SE)	(SE)	(SE)	(SE)	(SE)	
		[p-value]	[p-value]	[p-value]	[p-value]	
		<q-value>	<q-value>	<q-value>	<q-value>	

Autonomy in income	0.76	0.06	0.00	−0.01	0.07	2,739
	(0.03)	(0.04)	(0.04)	(0.04)	(0.04)	
		[0.08]*	[0.91]	[0.90]	[0.12]	
		<0.62>	<1.00>	<1.00>	<0.64>	
Self-efficacy	0.50	0.03	0.07	0.06	0.05	2,739
	(0.04)	(0.05)	(0.05)	(0.05)	(0.05)	
		[0.55]	[0.17]	[0.32]	[0.35]	
		<1.00>	<0.71>	<1.00>	<1.00>	
Attitudes about intimate partner violence against women	0.62	0.01	0.05	0.02	0.09	2,739
	(0.04)	(0.05)	(0.05)	(0.05)	(0.05)	
		[0.78]	[0.37]	[0.62]	[0.09]*	
		<1.00>	<1.00>	<1.00>	<0.62>	
Respect among household members	0.71	−0.02	0.01	0.05	0.01	2,739
	(0.04)	(0.04)	(0.04)	(0.05)	(0.04)	
		[0.65]	[0.84]	[0.33]	[0.81]	
		<1.00>	<1.00>	<1.00>	<1.00>	
Input in productive decisions	0.86	0.04	0.01	0.02	0.03	2,732
	(0.02)	(0.02)	(0.02)	(0.02)	(0.02)	
		[0.10]	[0.61]	[0.36]	[0.31]	
		<0.62>	<1.00>	<1.00>	<1.00>	
Ownership of land and other assets	0.81	0.03	−0.01	0.02	0.07	2,739
	(0.03)	(0.04)	(0.04)	(0.04)	(0.04)	
		[0.42]	[0.74]	[0.63]	[0.07]*	
		<1.00>	<1.00>	<1.00>	<0.62>	
Access to and decisions on financial services	0.62	0.20	0.23	0.22	0.19	2,732
	(0.02)	(0.03)	(0.03)	(0.03)	(0.03)	
		[0.00]***	[0.00]***	[0.00]***	[0.00]***	
		<0.00>†††	<0.00>†††	<0.00>†††	<0.00>†††	
Control over use of income	0.80	0.06	0.02	0.03	0.06	2,739
	(0.03)	(0.03)	(0.03)	(0.03)	(0.03)	
		[0.07]*	[0.52]	[0.38]	[0.07]*	
		<0.62>	<1.00>	<1.00>	<0.62>	
Work balance	0.48	−0.01	0.00	−0.02	0.01	2,739
	(0.03)	(0.05)	(0.05)	(0.05)	(0.05)	
		[0.89]	[0.96]	[0.76]	[0.82]	
		<1.00>	<1.00>	<1.00>	<1.00>	
Visiting important locations	0.24	0.02	0.08	0.04	0.07	2,739
	(0.03)	(0.05)	(0.05)	(0.05)	(0.05)	
		[0.66]	[0.10]*	[0.37]	[0.15]	
		<1.00>	<0.62>	<1.00>	<0.71>	
Group membership	0.45	0.03	−0.01	0.01	0.08	2,739
	(0.03)	(0.05)	(0.04)	(0.03)	(0.04)	
		[0.53]	[0.89]	[0.88]	[0.05]**	
		<1.00>	<1.00>	<1.00>	<0.62>	
Membership in influential groups	0.21	0.01	−0.03	0.03	0.03	2,739
	(0.02)	(0.04)	(0.04)	(0.04)	(0.04)	
		[0.71]	[0.51]	[0.45]	[0.48]	
		<1.00>	<1.00>	<1.00>	<1.00>	

*Note:* Estimates are intent-to-treat, and estimated as marginal effects for the 12 pro-WEAI indicators. Standard errors adjusted for clustering at block level are in parentheses. *p < .10; **p < .05; ***p < .01; †q < 0.10; ††q < 0.05; †††q < 0.01. p-values in square brackets; Anderson (2008) sharpened q-values in triangular brackets.

All specifications include as independent variables the treatment indicators, age and education level of both respondents, number of children (0–15 years) in the household, number of adults (15 + years) in the household, size of household operated land, a wealth index and upazila at baseline. See notes to Appendix Table 2 for the definition of treatment arms.

**Table A3b:** (Panel B): Single-difference impacts on pro-WEAI outcomes (Men).

		Impacts				Number of observations
Indicator	Control	T-N	T-A	T-AN	T-ANG	
	Mean	Coeff	Coeff	Coeff	Coeff	
	(SE)	(SE)	(SE)	(SE)	(SE)	
		[p-value]	[p-value]	[p-value]	[p-value]	
		<q-value>	<q-value>	<q-value>	<q-value>	

Autonomy in income	0.95	0.01	0.00	−0.03	−0.02	2,732
	(0.01)	(0.02)	(0.02)	(0.02)	(0.02)	
		[0.56]	[0.80]	[0.09]*	[0.30]	
		<1.00>	<1.00>	<1.00>	<1.00>	
Self-efficacy	0.60	0.06	0.04	0.01	−0.06	2,739
	(0.04)	(0.05)	(0.05)	(0.05)	(0.05)	
		[0.24]	[0.48]	[0.81]	[0.24]	
		<1.00>	<1.00>	<1.00>	<1.00>	
Attitudes about intimate partner violence against women	0.70	0.09	0.02	0.02	0.04	2,739
	(0.03)	(0.04)	(0.04)	(0.04)	(0.04)	
		[0.02]**	[0.52]	[0.57]	[0.36]	
		<0.30>	<1.00>	<1.00>	<1.00>	
Respect among household members	0.82	−0.01	−0.02	−0.04	−0.06	2,732
	(0.03)	(0.05)	(0.05)	(0.05)	(0.05)	
		[0.78]	[0.71]	[0.43]	[0.23]	
		<1.00>	<1.00>	<1.00>	<1.00>	
Input in productive decisions	0.90	−0.00	−0.01	−0.03	0.01	2,739
	(0.02)	(0.02)	(0.03)	(0.03)	(0.03)	
		[0.86]	[0.62]	[0.24]	[0.77]	
		<1.00>	<1.00>	<1.00>	<1.00>	
Ownership of land and other assets	0.99	0.00	−0.01	−0.00	−0.00	2,739
	(0.00)	(0.01)	(0.01)	(0.01)	(0.01)	
		[0.55]	[0.20]	[0.37]	[0.50]	
		<1.00>	<1.00>	<1.00>	<1.00>	
Access to and decisions on financial services	0.78	0.11	0.10	0.04	0.07	2,739
	(0.02)	(0.03)	(0.03)	(0.03)	(0.03)	
		[0.00]***	[0.00]***	[0.16]	[0.02]**	
		<0.02>††	<0.02>††	<1.00>	<0.32>	
Control over use of income	0.86	−0.00	−0.03	−0.03	0.00	2,739
	(0.02)	(0.04)	(0.03)	(0.03)	(0.04)	
		[0.94]	[0.41]	[0.40]	[0.90]	
		<1.00>	<1.00>	<1.00>	<1.00>	
Work balance	0.53	−0.05	−0.04	0.02	0.01	2,739
	(0.02)	(0.04)	(0.04)	(0.04)	(0.04)	
		[0.20]	[0.39]	[0.61]	[0.85]	
		<1.00>	<1.00>	<1.00>	<1.00>	
Visiting important locations	0.49	0.09	0.04	0.03	0.02	2,739
	(0.03)	(0.05)	(0.05)	(0.05)	(0.06)	
		[0.05]**	[0.38]	[0.50]	[0.69]	
		<0.71>	<1.00>	<1.00>	<1.00>	
Group membership	0.29	0.07	0.01	0.02	0.06	2,739
	(0.03)	(0.04)	(0.05)	(0.04)	(0.04)	
		[0.12]	[0.88]	[0.60]	[0.19]	
		<1.00>	<1.00>	<1.00>	<1.00>	
Membership in influential groups	0.17	0.02	−0.01	−0.02	−0.02	2,739
	(0.02)	(0.03)	(0.03)	(0.03)	(0.03)	
		[0.38]	[0.84]	[0.55]	[0.55]	
		<1.00>	<1.00>	<1.00>	<1.00>	

*Note:* Estimates are intent-to-treat, and estimated as marginal effects for the 12 pro-WEAI indicators. Standard errors adjusted for clustering at block level are in parentheses. *p < .10; **p < .05; ***p < .01; †q < 0.10; ††q < 0.05; †††q < 0.01. p-values in square brackets; Anderson (2008) sharpened q-values in triangular brackets.

See notes to Appendix Table 3 for specifications and notes to Appendix Table 2 for definition of treatment arms.

**Table A4a:** (Panel A): Single-difference impacts on gender attitudes outcomes (Women).

		Impacts				Number of observations
Indicator	Control	T-N	T-A	T-AN	T-ANG	
	Mean	Coeff	Coeff	Coeff	Coeff	
	(SE)	(SE)	(SE)	(SE)	(SE)	
		[p-value]	[p-value]	[p-value]	[p-value]	
		<q-value>	<q-value>	<q-value>	<q-value>	

I make important contributions to my family	4.68	0.02	0.01	0.03	−0.04	2,739
	(0.03)	(0.05)	(0.06)	(0.05)	(0.05)	
		[0.69]	[0.89]	[0.60]	[0.45]	
		<1.00>	<1.00>	<1.00>	<1.00>	
Some adults in my family do not make important contributions to the family	2.40	−0.01	0.01	−0.10	−0.04	2,739
	(0.09)	(0.17)	(0.16)	(0.16)	(0.14)	
		[0.97]	[0.95]	[0.50]	[0.74]	
		<1.00>	<1.00>	<1.00>	<1.00>	
I make important contributions to my community	3.23	−0.04	0.15	0.21	0.18	2,739
	(0.07)	(0.13)	(0.11)	(0.12)	(0.12)	
		[0.77]	[0.19]	[0.09]*	[0.13]	
		<1.00>	<1.00>	<1.00>	<1.00>	
I sometimes refrain from voicing my opinion because I fear being ignored/ridiculed	3.46	−0.00	−0.07	−0.19	−0.20	2,739
	(0.08)	(0.10)	(0.13)	(0.12)	(0.13)	
		[0.97]	[0.60]	[0.12]	[0.12]	
		<1.00>	<1.00>	<1.00>	<1.00>	
I have a hard time saying positive things about myself	3.51	−0.01	−0.01	−0.08	−0.05	2,739
	(0.07)	(0.10)	(0.13)	(0.13)	(0.10)	
		[0.96]	[0.94]	[0.52]	[0.59]	
		<1.00>	<1.00>	<1.00>	<1.00>	
Women should stand up for themselves to get what they want	4.67	0.07	0.02	0.06	0.05	2,739
	(0.03)	(0.05)	(0.05)	(0.04)	(0.04)	
		[0.13]	[0.68]	[0.14]	[0.28]	
		<1.00>	<1.00>	<1.00>	<1.00>	
Women are usually very busy with work that benefits the household	4.74	−0.02	0.01	0.01	0.02	2,739
	(0.03)	(0.05)	(0.04)	(0.04)	(0.04)	
		[0.75]	[0.79]	[0.73]	[0.59]	
		<1.00>	<1.00>	<1.00>	<1.00>	
Husbands should help wives with household chores like cooking and taking care of children	4.62	0.07	0.02	0.09	0.05	2,739
	(0.04)	(0.06)	(0.05)	(0.05)	(0.05)	
		[0.29]	[0.67]	[0.08]*	[0.33]	
		<1.00>	<1.00>	<1.00>	<1.00>	
We can change culture/tradition regarding what men/women do and how they relate to each other	3.87	0.20	−0.02	0.16	0.24	2,739
	(0.10)	(0.12)	(0.13)	(0.14)	(0.13)	
		[0.11]	[0.90]	[0.25]	[0.05]*	
		<1.00>	<1.00>	<1.00>	<1.00>	

*Note:* Estimates are intent-to-treat. Standard errors adjusted for clustering at block level are in parentheses. *p < .10; **p < .05; ***p < .01; †q < 0.10; ††q < 0.05; †††q < 0.01. p-values in square brackets; Anderson (2008) sharpened q-values in triangular brackets.

See notes to Appendix Table 3 for specifications and notes to Appendix Table 2 for definition of treatment arms.

**Table A4b:** (Panel B): Single-difference impacts on gender attitudes outcomes (Men).

		Impacts				Number of observations
Indicator	Control	T-N	T-A	T-AN	T-ANG	
	Mean	Coeff	Coeff	Coeff	Coeff	
	(SE)	(SE)	(SE)	(SE)	(SE)	
		[p-value]	[p-value]	[p-value]	[p-value]	
		<q-value>	<q-value>	<q-value>	<q-value>	

I make important contributions to my family	4.60	0.09	0.13	0.06	0.06	2,739
	(0.04)	(0.05)	(0.05)	(0.06)	(0.06)	
		[0.05]*	[0.01]**	[0.34]	[0.31]	
		<0.27>	<0.24>	<0.76>	<0.76>	
Some adults in my family do not make important contributions to the family	2.46	−0.09	−0.16	−0.22	−0.08	2,739
	(0.09)	(0.12)	(0.10)	(0.12)	(0.13)	
		[0.48]	[0.11]	[0.07]*	[0.56]	
		<0.80>	<0.37>	<0.31>	<0.82>	
I make important contributions to my community	3.77	0.11	0.04	0.13	−0.08	2,739
	(0.06)	(0.08)	(0.08)	(0.09)	(0.10)	
		[0.17]	[0.61]	[0.15]	[0.41]	
		<0.47>	<0.82>	<0.45>	<0.79>	
I sometimes refrain from voicing my opinion because I fear being ignored/ridiculed	3.19	−0.09	−0.18	−0.17	−0.30	2,739
	(0.08)	(0.12)	(0.10)	(0.13)	(0.13)	
		[0.48]	[0.08]*	[0.19]	[0.02]**	
		<0.80>	<0.32>	<0.48>	<0.25>	
I have a hard time saying positive things about myself	3.36	−0.05	−0.10	−0.05	−0.17	2,739
	(0.08)	(0.12)	(0.11)	(0.12)	(0.12)	
		[0.67]	[0.36]	[0.68]	[0.16]	
		<0.82>	<0.76>	<0.82>	<0.45>	
Women should stand up for themselves to get what they want	4.45	−0.04	−0.09	0.01	0.04	2,739
	(0.05)	(0.06)	(0.07)	(0.06)	(0.06)	
		[0.53]	[0.23]	[0.85]	[0.58]	
		<0.81>	<0.58>	<1.00>	<0.82>	
Women are usually very busy with work that benefits the household	4.51	0.01	−0.05	0.09	0.10	2,739
	(0.04)	(0.05)	(0.06)	(0.05)	(0.05)	
		[0.79]	[0.36]	[0.05]*	[0.04]**	
		<0.95>	<0.76>	<0.27>	<0.27>	
Husbands should help wives with household chores like cooking and taking care of children	4.42	0.10	0.03	0.12	0.17	2,739
	(0.04)	(0.05)	(0.06)	(0.05)	(0.05)	
		[0.05]**	[0.63]	[0.02]**	[0.00]***	
		<0.27>	<0.82>	<0.25>	<0.03>††	
We can change culture/tradition regarding what men/women do and how they relate to each other	3.74	0.07	−0.03	0.04	0.01	2,739
	(0.07)	(0.10)	(0.11)	(0.09)	(0.10)	
		[0.46]	[0.75]	[0.66]	[0.89]	
		<0.80>	<0.90>	<0.82>	<1.00>	

*Note:* Estimates are intent-to-treat. Standard errors adjusted for clustering at block level are in parentheses. *p < .10; **p < .05; ***p < .01; †q < 0.10; ††q < 0.05; †††q < 0.01. p-values in square brackets; Anderson (2008) sharpened q-values in triangular brackets.

See notes to Appendix Table 3 for specifications and notes to Appendix Table 2 for definition of treatment arms.

**Table A5a:** (Panel A): Single-difference impacts on IPV experience outcomes (Women).

		Impacts				Number of observations
Indicator	Control	T-N	T-A	T-AN	T-ANG	
	Mean	Coeff	Coeff	Coeff	Coeff	
	(SE)	(SE)	(SE)	(SE)	(SE)	
		[p-value]	[p-value]	[p-value]	[p-value]	
		<q-value>	<q-value>	<q-value>	<q-value>	

*Experience of violence in the past six months*						
Insulted you or made you feel bad about yourself	0.20	−0.01	0.02	−0.02	0.07	2,739
	(0.03)	(0.04)	(0.05)	(0.04)	(0.04)	
		[0.82]	[0.66]	[0.63]	[0.13]	
		<1.00>	<1.00>	<1.00>	<1.00>	
Belittled or humiliated you in front of other people	0.10	−0.01	0.00	0.02	0.05	2,732
	(0.02)	(0.03)	(0.03)	(0.03)	(0.03)	
		[0.75]	[0.99]	[0.56]	[0.12]	
		<1.00>	<1.00>	<1.00>	<1.00>	
Done things to scare or intimidate you on purpose	0.05	−0.00	0.02	0.02	0.05	2,732
	(0.01)	(0.02)	(0.02)	(0.02)	(0.02)	
		[0.92]	[0.42]	[0.38]	[0.02]**	
		<1.00>	<1.00>	<1.00>	<1.00>	
Threatened to hurt you or someone you care about	0.00	0.00	−0.00	0.00	0.01	2,732
	(0.00)	(0.00)	(0.01)	(0.00)	(0.01)	
		[0.87]	[0.70]	[0.48]	[0.10]*	
		<1.00>	<1.00>	<1.00>	<1.00>	
Slapped you or thrown something at you that could hurt you	0.06	−0.01	0.02	−0.02	0.02	2,725
	(0.01)	(0.02)	(0.02)	(0.02)	(0.02)	
		[0.51]	[0.39]	[0.38]	[0.22]	
		<1.00>	<1.00>	<1.00>	<1.00>	
Pushed you or shoved you or pulled your hair	0.02	0.01	0.01	0.01	0.01	2,727
	(0.01)	(0.01)	(0.01)	(0.01)	(0.01)	
		[0.42]	[0.18]	[0.44]	[0.42]	
		<1.00>	<1.00>	<1.00>	<1.00>	
Hit you with his fist or with something else that could hurt you	0.01	0.00	0.01	0.00	0.01	2,720
	(0.00)	(0.01)	(0.01)	(0.01)	(0.01)	
		[0.59]	[0.45]	[0.68]	[0.30]	
		<1.00>	<1.00>	<1.00>	<1.00>	
Kicked you, dragged you or beat you up	0.01	0.00	−0.01	0.00	0.01	2,718
	(0.00)	(0.01)	(0.01)	(0.01)	(0.01)	
		[0.60]	[0.60]	[0.66]	[0.08]*	
		<1.00>	<1.00>	<1.00>	<1.00>	
Choked or burnt you on purpose	0.00	−0.00	−0.00	0.00	0.01	2,718
	(0.00)	(0.01)	(0.01)	(0.00)	(0.00)	
		[0.44]	[0.46]	[0.69]	[0.11]	
		<1.00>	<1.00>	<1.00>	<1.00>	

*Note:* Estimates are intent-to-treat, and estimated as marginal effects. Standard errors adjusted for clustering at block level are in parentheses. *p < .10; **p < .05; ***p < .01; †q < 0.10; ††q < 0.05; †††q < 0.01. p-values in square brackets; Anderson (2008) sharpened q-values in triangular brackets.

See notes to Appendix Table 3 for specifications and notes to Appendix Table 2 for definition of treatment arms.

**Table A5b:** (Panel B): Single-difference impacts on IPV frequency outcomes (Women).

		Impacts				Number of observations
Indicator	Control	T-N	T-A	T-AN	T-ANG	
	Mean	Coeff	Coeff	Coeff	Coeff	
	(SE)	(SE)	(SE)	(SE)	(SE)	
		[p-value]	[p-value]	[p-value]	[p-value]	
		<q-value>	<q-value>	<q-value>	<q-value>	

*Frequency of violence in the past six months*						
Insulted you or made you feel bad about yourself	0.35	−0.01	0.02	−0.02	0.07	2,739
	(0.05)	(0.04)	(0.05)	(0.04)	(0.04)	
		[0.82]	[0.66]	[0.63]	[0.13]	
		<1.00>	<1.00>	<1.00>	<1.00>	
Belittled or humiliated you in front of other people	0.18	−0.01	0.00	0.02	0.05	2,732
	(0.03)	(0.03)	(0.03)	(0.03)	(0.03)	
		[0.75]	[0.99]	[0.56]	[0.12]	
		<1.00>	<1.00>	<1.00>	<1.00>	
Done things to scare or intimidate you on purpose	0.10	−0.00	0.02	0.02	0.05	2,732
	(0.02)	(0.02)	(0.02)	(0.02)	(0.02)	
		[0.92]	[0.42]	[0.38]	[0.02]**	
		<1.00>	<1.00>	<1.00>	<1.00>	
Threatened to hurt you or someone you care about	0.01	0.00	−0.00	0.00	0.01	2,732
	(0.00)	(0.00)	(0.01)	(0.00)	(0.01)	
		[0.87]	[0.70]	[0.48]	[0.10]*	
		<1.00>	<1.00>	<1.00>	<1.00>	
Slapped you or thrown something at you that could hurt you	0.09	−0.01	0.02	−0.02	0.02	2,725
	(0.02)	(0.02)	(0.02)	(0.02)	(0.02)	
		[0.51]	[0.39]	[0.38]	[0.22]	
		<1.00>	<1.00>	<1.00>	<1.00>	
Pushed you or shoved you or pulled your hair	0.02	0.01	0.01	0.01	0.01	2,727
	(0.01)	(0.01)	(0.01)	(0.01)	(0.01)	
		[0.42]	[0.18]	[0.44]	[0.42]	
		<1.00>	<1.00>	<1.00>	<1.00>	
Hit you with his fist or with something else that could hurt you	0.02	0.00	0.01	0.00	0.01	2,720
	(0.01)	(0.01)	(0.01)	(0.01)	(0.01)	
		[0.59]	[0.45]	[0.68]	[0.30]	
		<1.00>	<1.00>	<1.00>	<1.00>	
Kicked you, dragged you or beat you up	0.01	0.00	−0.01	0.00	0.01	2,718
	(0.01)	(0.01)	(0.01)	(0.01)	(0.01)	
		[0.60]	[0.60]	[0.66]	[0.08]*	
		<1.00>	<1.00>	<1.00>	<1.00>	
Choked or burnt you on purpose	0.01	−0.00	−0.00	0.00	0.01	2,718
	(0.00)	(0.01)	(0.01)	(0.00)	(0.00)	
		[0.44]	[0.46]	[0.69]	[0.11]	
		<1.00>	<1.00>	<1.00>	<1.00>	

*Note:* Estimates are intent-to-treat. Standard errors adjusted for clustering at block level are in parentheses. *p < .10; **p < .05; ***p < .01; †q < 0.10; ††q < 0.05; †††q < 0.01. p-values in square brackets; Anderson (2008) sharpened q-values in triangular brackets.

See notes to Appendix Table 3 for specifications and notes to Appendix Table 2 for definition of treatment arms.

**Table A6a:** (Panel A): Single-difference impacts on attitudes about intimate partner violence against women (Women).

		Impacts				Number of observations
Indicator	Control	T-N	T-A	T-AN	T-ANG	
	Mean	Coeff	Coeff	Coeff	Coeff	
	(SE)	(SE)	(SE)	(SE)	(SE)	
		[p-value]	[p-value]	[p-value]	[p-value]	
		<q-value>	<q-value>	<q-value>	<q-value>	

*Proportion agreeing that violence not justified if woman*						
goes out without telling husband	0.81	0.04	0.05	0.03	0.07	2,739
	(0.03)	(0.03)	(0.04)	(0.04)	(0.04)	
		[0.18]	[0.23]	[0.34]	[0.07]*	
		<1.00>	<1.00>	<1.00>	<1.00>	
neglects the children	0.76	0.04	0.05	0.06	0.06	2,739
	(0.03)	(0.04)	(0.04)	(0.04)	(0.04)	
		[0.37]	[0.20]	[0.20]	[0.19]	
		<1.00>	<1.00>	<1.00>	<1.00>	
argues with husband	0.70	−0.01	0.01	0.01	0.10	2,739
	(0.03)	(0.04)	(0.04)	(0.04)	(0.05)	
		[0.84]	[0.76]	[0.83]	[0.03]**	
		<1.00>	<1.00>	<1.00>	<1.00>	
refuses to have sex with husband	0.90	0.00	0.02	−0.02	0.02	2,738
	(0.02)	(0.03)	(0.02)	(0.03)	(0.03)	
		[0.90]	[0.32]	[0.58]	[0.41]	
		<1.00>	<1.00>	<1.00>	<1.00>	
burns the food	0.93	−0.01	−0.00	−0.00	0.02	2,739
	(0.01)	(0.02)	(0.02)	(0.02)	(0.02)	
		[0.66]	[0.95]	[0.98]	[0.44]	
		<1.00>	<1.00>	<1.00>	<1.00>	

*Note:* Estimates are intent-to-treat, and estimated as marginal effects. Standard errors adjusted for clustering at block level are in parentheses. *p < .10; **p < .05; ***p < .01; †q < 0.10; ††q < 0.05; †††q < 0.01. p-values in square brackets; Anderson (2008) sharpened q-values in triangular brackets.

See notes to Appendix Table 3 for specifications and notes to Appendix Table 2 for definition of treatment arms. Respondents were asked whether a husband was justified in hitting or beating his wife in the following situations: if she goes out without telling him; if she neglects the children; if she argues with him; if she refuses to have sex with him; if she burns the food. Response options were Yes, No, Don’t Know. Variables have been reverse coded in this table reflecting that a husband is not justified in beating his wife in each situation.

**Table A6b:** (Panel B): Single-difference impacts on attitudes about intimate partner violence against women (Men).

		Impacts				Number of observations
Indicator	Control	T-N	T-A	T-AN	T-ANG	
	Mean	Coeff	Coeff	Coeff	Coeff	
	(SE)	(SE)	(SE)	(SE)	(SE)	
		[p-value]	[p-value]	[p-value]	[p-value]	
		<q-value>	<q-value>	<q-value>	<q-value>	

*Proportion agreeing that violence not justified if woman*						
goes out without telling husband	0.87	0.04	0.01	−0.01	−0.01	2,739
	(0.02)	(0.02)	(0.03)	(0.03)	(0.03)	
		[0.10]*	[0.69]	[0.78]	[0.68]	
		<1.00>	<1.00>	<1.00>	<1.00>	
neglects the children	0.86	0.01	−0.01	−0.01	−0.04	2,739
	(0.02)	(0.03)	(0.03)	(0.03)	(0.03)	
		[0.83]	[0.74]	[0.65]	[0.23]	
		<1.00>	<1.00>	<1.00>	<1.00>	
argues with husband	0.77	0.09	0.02	0.03	0.06	2,739
	(0.03)	(0.03)	(0.03)	(0.03)	(0.03)	
		[0.00]***	[0.60]	[0.32]	[0.08]*	
		<0.06>†	<1.00>	<1.00>	<1.00>	
refuses to have sex with husband	0.95	0.02	−0.01	−0.00	0.01	2,737
	(0.01)	(0.02)	(0.01)	(0.02)	(0.02)	
		[0.32]	[0.62]	[0.97]	[0.49]	
		<1.00>	<1.00>	<1.00>	<1.00>	
burns the food	0.94	0.01	−0.00	0.02	0.02	2,732
	(0.01)	(0.02)	(0.02)	(0.02)	(0.02)	
		[0.44]	[0.93]	[0.15]	[0.21]	
		<1.00>	<1.00>	<1.00>	<1.00>	

*Note:* Estimates are intent-to-treat, and estimated as marginal effects. Standard errors adjusted for clustering at block level are in parentheses. *p < .10; **p < .05; ***p < .01; †q < 0.10; ††q < 0.05; †††q < 0.01. p-values in square brackets; Anderson (2008) sharpened q-values in triangular brackets.

See notes to Appendix Table 6 (Panel A for specifications and definitions of variables).

**Table A7:** Double-difference impacts on A-WEAI outcomes.

		Impacts				Test of difference between arms				Number of observations
Indicator	Control	T-N	T-A	T-AN	T-ANG	T-N = T-A	T-N = T-AN	T-A = T-AN	T-AN = T-ANG	
	Mean					p-value				
	(SE)									

Women										
A-WEAI: Empowerment score	0.79	0.00	−0.00	−0.01	0.02	0.65	0.54	0.93	0.09*	2,739
	(0.01)	(0.01)	(0.01)	(0.01)	(0.01)					
A-WEAI: Whether empowered	0.67	−0.01	−0.01	0.02	0.04	0.96	0.52	0.50	0.49	2,732
	(0.02)	(0.04)	(0.04)	(0.04)	(0.03)					

Men										
A-WEAI: Empowerment score	0.76	0.01	−0.00	0.01	0.02	0.49	0.82	0.39	0.74	2,739
	(0.01)	(0.01)	(0.01)	(0.01)	(0.01)					
A-WEAI: Whether empowered	0.65	0.02	0.00	0.04	0.04	0.75	0.63	0.44	0.96	2,739
	(0.03)	(0.04)	(0.04)	(0.04)	(0.04)					

Household										
A-WEAI: HH achieves gender parity	0.75	−0.02	−0.00	0.00	0.02	0.62	0.53	0.87	0.67	2,732
	(0.02)	(0.03)	(0.03)	(0.03)	(0.03)					

*Note:* Estimates are intent-to-treat, and estimated as marginal effects for whether empowered and gender parity indicators. The empowerment score is the weighted average of the six A-WEAI indicators. An individual is defined as empowered if s/he reaches the threshold of 80 percent or more of the weighted indicators. A household achieves gender parity if the woman respondent is empowered or her empowerment score is equal to or greater than that of the man respondent in the household. Standard errors adjusted for clustering at block level are in parentheses. *p < .10; **p < .05; ***p < .01.

See notes to Appendix Table 3 for specifications and notes to Appendix Table 2 for definition of treatment arms.

## Footnotes

1 This section draws on Ahmed et al. (2018).

2 There was an additional nutrition BCC treatment arm that used community women rather than agricultural extension agents (AEAs) to deliver the nutrition intervention. It is not used in this analysis, both because of less practical relevance (the Ministry of Agriculture plans to use its nationwide agricultural extension workforce to expand ANGeL across the country) and because it was not part of the bundled interventions that we compare to understand additive effects.

3 The curriculum and materials for the agricultural production training were developed by Helen Keller International (HKI) in collaboration with the Bangladesh Agricultural Research Institute (BARI) and the Bangladesh Rice Research Institute (BRRI).

4 HKI developed the curriculum and training materials for the nutrition BCC with the Bangladesh Institute of Research and Training on Applied Nutrition (BIRTAN) and the International Food Policy Research Institute. HKI was responsible for training the AEAs.

5 These data are based on self-reports; for example, women provided information on their own attendance. We also asked respondents to provide information on the attendance of their spouses; this produced similar patterns to that reported here.

6 We received permission from the Ministry of Agriculture, Government of Bangladesh who issued Letters of Authorization to conduct the surveys described below. The surveys received ethical approval from the Institutional Review Board of the International Food Policy Research Institute (IRB approval number 00007490). The study was registered on the Registry for International Development Evaluations (RIDIE-STUDY-ID-5afbe43292b4c).
