# Supplementary material for: Designing for empowerment impact in agricultural development projects: Experimental evidence from the Agriculture, Nutrition, and Gender Linkages (ANGeL) project in Bangladesh
Source: World Dev. 2021 Oct;146:105622. doi: 10.1016/j.worlddev.2021.105622 (PMC8350314; doi:10.1016/j.worlddev.2021.105622)
Supplement: Supplementary Data 1 [file mmc1.docx]

The project-level Women’s Empowerment in Agriculture Index (pro-WEAI):

Summary and questionnaire

This supplementary material draws heavily from Malapit et al. (2019), which is published as an open-access article in this journal. Questionnaires, enumerator manuals, qualitative protocols, and do-files on pro-WEAI and WEAI are available at the WEAI Resource Center, www.weai.ifpri.info.

## Domains and indicators of pro-WEAI

Pro-WEAI is an adaptation of the Women’s Empowerment in Agricultural Index (WEAI) (Alkire et al. 2013) for use by agricultural development projects (see Malapit et al. 2019) for details. It was jointly developed by IFPRI and 13 agricultural development projects as part of the Gender, Agriculture, and Assets Project, Phase 2 (GAAP2 for pro-WEAI). Both the WEAI and pro-WEAI are rooted in Kabeer’s (1999, 2005) framework of empowerment, which describes empowerment as a process of change on the interrelated dimensions of resources, agency, and achievements, and focuses on measuring agency, or the ability of individuals to make strategic choices.

Whereas the original WEAI had five domains of empowerment with 10 indicators organized thematically and aligned with FTF programming priorities, pro-WEAI has 12 indicators mapped to three domains: intrinsic agency (power within), instrumental agency (power to), and collective agency (power with). These three aspects of agency reflect the generative types of power described above (Rowlands 1997; Ibrahim and Alkire 2007) and are present in the earlier WEAI, although not explicitly. These theoretical links are strengthened in the pro-WEAI.

Table S1 presents full definitions for the pro-WEAI indicators and, if the indicator was previously included in the WEAI, how the pro-WEAI indicator differs. The four indicators of intrinsic agency include autonomy in income, self-efficacy, attitudes about IPV against women,^8^ and respect among household members. The six indicators of instrumental agency include input into productive decisions, ownership of land and other assets, control over use of income, access to and decisions on financial services, workload, and visiting important locations. Collective agency is comprised of group membership and membership in influential groups. Seven out of the 12 indicators in pro-WEAI are adapted from the original WEAI indicators, ^9^ and five indicators are new (attitudes about IPV against women, self-efficacy, respect among household members, visiting important locations, membership in influential groups) and stem from topics that the projects themselves suggested. Each indicator is equally weighted, and a person is defined as empowered if she or he is empowered in at least nine of 12 indicators, or 75 percent.

The WEAI and pro-WEAI rely heavily on instrumental agency indicators, comprised mainly of decision-making questions. Decision-making questions are often used in surveys, and span many different aspects (e.g., production, assets, credit, etc.), so these questions have been tested and used more widely than have indicators on intrinsic and collective agency. The reliance on instrumental agency implies that households with only female decision-makers are more likely to be identified as empowered by default, which is a known limitation of WEAI (Alkire et al. 2013). While several aspects of instrumental agency are well established in the theoretical literature, we had a smaller pool of candidate indicators to draw on for measuring intrinsic and collective agency in developing pro-WEAI. ^10^

*Table S1. Pro-WEAI indicators, definitions of adequacy, and comparison to the original WEAI*

| **Indicator** ^A^ | **Definition of adequacy** | **Difference compared to original WEAI** |
| --- | --- | --- |
| ***Intrinsic Agency*** | | |
| Autonomy in  income | More motivated by own values than by coercion or fear of others’ disapproval: *Relative Autonomy Index* ^B^ score>=1  RAI score is calculated by summing responses to the three vignettes about a person’s motivation for how they use income generated from agricultural and non-agricultural activities (yes=1; no=0), using the following weighting scheme: 0 for vignette 1 (no alternative), -2 for vignette 2 (external motivation), -1 for vignette 3 (introjected motivation), and +3 for vignette 4 (autonomous motivation) | Based on “Autonomy in production” indicator in the WEAI but now focuses exclusively on the use of income generated from agricultural and non-agricultural activities and uses a new vignette-based survey instrument. |
| Self-efficacy | "Agree" or greater on average with self-efficacy questions: *New General Self-Efficacy Scale* ^C^ score>=32 | Not included in the WEAI |
| Attitudes about intimate partner violence against women | Believes husband is NOT justified in hitting or beating his wife in all 5 scenarios: ^D^   1. She goes out without telling him 2. She neglects the children 3. She argues with him 4. She refuses to have sex with him 5. She burns the food | Not included in the WEAI |
| Respect among household members | Meets ALL of the following conditions related to their spouse, the other respondent, or another household member:   1. Respondent respects relation (MOST of the time) AND 2. Relation respects respondent (MOST of the time) AND 3. Respondent trusts relation (MOST of the time) AND 4. Respondent is comfortable disagreeing with relation (MOST of the time) | Not included in the WEAI |
| ***Instrumental Agency*** | | |
| Input in productive decisions | Meets at least ONE of the following conditions for ALL of the agricultural activities they participate in   1. Makes related decision solely, 2. Makes the decision jointly and has at least some input into the decisions 3. Feels could make decision if wanted to (to at least a MEDIUM extent) | Included in the WEAI, but now uses a stricter adequacy cut-off |
| Ownership of land and other assets | Owns, either solely or jointly, at least ONE of the following:   1. At least THREE small assets (poultry, nonmechanized equipment, or small consumer durables) 2. At least TWO large assets 3. Land | Included in the WEAI, but now uses a stricter adequacy cut-off |
| Access to and decisions on financial services | Meets at least ONE of the following conditions:   1. Belongs to a household that used a source of credit in the past year AND participated in at least ONE sole or joint decision about it 2. Belongs to a household that did not use credit in the past year but could have if wanted to from at least ONE source 3. Has access, solely or jointly, to a financial account | Based on “Access to and decisions on credit” indicator in the WEAI, but now includes access to financial accounts |
| Control over use of income | Has input in decisions related to how to use BOTH income and output from ALL of the agricultural activities they participate in AND has input in decisions related to income from ALL non-agricultural activities they participate in, unless no decision was made | Included in the WEAI, but now uses a stricter adequacy cut-off |
| Work balance | Works less than 10.5 hours per day:  Workload = time spent in primary activity + (1/2) time spent in childcare as a secondary activity | Similar to ‘Workload” indicator in the WEAI but restricts the measurement of secondary activities to a single activity: childcare. |
| Visiting important locations | Meets at least ONE of the following conditions:   1. Visits at least TWO locations at least ONCE PER WEEK of [city, market, family/relative], or 2. Visits least ONE location at least ONCE PER MONTH of [health facility, public meeting] | Not included in the WEAI |
| ***Collective Agency*** | | |
| Group membership | Active member of at least ONE group | Same as in the WEAI |
| Membership in influential groups | Active member of at least ONE group that can influence the community to at least a MEDIUM extent | Not included in the WEAI |

**Notes:** ^A^ All indicators are equally weighted (1/12) in the pro-WEAI.

^B^ The Relative Autonomy Index (RAI), based on self-determination theory, is a measure of internal and external motivations that determine person’s decisions (Ryan and Deci 2000). The text for vignettes 1-4 can be found in Appendix C, module G8(A).

^C^ The New General Self-efficacy Scale (NGSE) is a validated scale to measure self-efficacy, or a person’s capabilities and ability to reach their goals (Chen, Gully and Eden 2001). The questions can be found in Appendix C, module G8(B).

These scenarios are based on previously validated items from the Demographic and Health Surveys (Yount et al. 2014).

## 2. Computation of the index

Each respondent in the pro-WEAI is classified as either adequate (=1) or inadequate (=0) in a given indicator by comparing their responses to the survey questions with a given threshold (Table S1). A respondent’s empowerment score is simply the weighted average of her/his adequacy scores in the 12 indicators (all weighted 1/12). If her/his score is 75% or higher, or if s/he is adequate in nine out of 12 indicators, then s/he is classified as empowered. Conversely, if her/his score is below 75%, or if s/he is inadequate in 4 or more indicators, then s/he is classified as disempowered. These individual level scores are then aggregated to construct pro-WEAI.

Pro-WEAI, similar to the original WEAI, is calculated as the weighted mean of two sub-indices: the Three Domains of Empowerment Index (3DE), with a weight of 90 percent, and the GPI, with a weight of 10 percent. The 3DE measures women’s empowerment across three domains: **intrinsic agency** (power within), **instrumental agency** (power to), and **collective agency** (power with). The GPI compares the empowerment scores of the eligible individual and her spouse, or the male respondent, in each household. The choice of weights for the two sub-indices follows the original WEAI, placing greater emphasis on the 3DE while still recognizing the importance of gender equality as an aspect of empowerment. Improvements in either the 3DE or GPI will increase pro-WEAI scores. While the aggregate pro-WEAI index, 5DE for women, 5DE for men, and GPI are all useful ways to summarize empowerment at the project level, we recommend interpreting these high-level indexes together with the sub-indicators, and sub-components. The decomposability of the index allows the user to disaggregate the drivers of change, and examine how women’s and men’s empowerment scores are contributing to it. Details on how the individual indicators are combined to form the pro-WEAI index are presented in Appendix B. -Dick et al. 2019).

Supplement Appendix A. Computation of the index

Computation of the pro-WEAI follows the methodology of the original WEAI (Alkire et al. 2013). Pro-WEAI is calculated as the weighted mean of two sub-indices: the Three Domains of Empowerment Index (3DE), with a weight of 90 percent, and the Gender Parity Index (GPI), with a weight of 10 percent. The 3DE measures women’s empowerment across three domains: **intrinsic agency** (power within), **instrumental agency** (power to), and **collective agency** (power with). The GPI compares the empowerment scores of the eligible individual and her spouse, or the male respondent, in each household. The choice of weights for the two sub-indices follows the original WEAI, placing greater emphasis on the 3DE while still recognizing the importance of gender equality as an aspect of empowerment. Improvements in either the 3DE or GPI will increase pro-WEAI scores.

### Supplement Appendix A.1 Three Domains of Empowerment Index (3DE)

To measure empowerment, we focus on the areas of disempowerment that must be overcome. We start by computing an index of disempowerment, $M_{0}$, using the Alkire-Foster method—an axiomatic and counting-based approach designed originally for measuring multidimensional poverty (Alkire and Foster 2011). This index captures the percentage of women who are disempowered, as well as the average share of inadequacies that they experience. This index varies between 0, when no one is disempowered, and 1, when everyone is disempowered and inadequate in all indicators. The 3DE is defined as (1 – $M_{0}$). This approach focuses on disempowered women and allows us to identify the key issues that need to be addressed to increase empowerment. We describe below the steps to compute the 3DE using a notation consistent with the $M_{0}$ measurement (Alkire and Foster 2011).

(i) Identify inadequacies. For each of the 12 indicators described in the previous section, a person is identified as adequate or inadequate. Person $i$ is inadequate in indicator $j$ if his or her level of achievement, $x_{ij}$, is below the adequacy cut-off $z_{j}$. To each person in each indicator, we assign an inadequacy status $g_{ij}=1$, if $x_{ij}<z_{j}$, and $g_{ij}=0$, otherwise.

(ii) Create the inadequacy score. For each person, the inadequacy score, $c_{i}$, is calculated by summing the inadequacy status of all indicators, each multiplied by their corresponding weight ${(w}_{j})$. More formally, $c_{i}=\sum_{j=1}^{12} w_{j}\times g_{ij}$. In pro-WEAI, all 12 indicators are equally weighted, and thus $w_{j}=1/12$. The inadequacy score represents the share of indicators in which a person is inadequate.

(iii) Identify the disempowered. To identify who is disempowered, we compare a person’s inadequacy score with the disempowerment cut-off, $k\in\left( 0,1 \right]$. The disempowerment cut-off is the share of (weighted) inadequacies an individual must have to be considered disempowered*.* Thus, a person is identified as disempowered if $c_{i}>k$, and empowered, otherwise.^[[1]](#footnote-2)^ In pro-WEAI, $k$ is set at 0.25, and thus a person is identified as disempowered if they are inadequate in at least 4 of the 12 indicators.

(iv) Compute the disempowerment headcount ratio. The disempowerment headcount ratio or the percentage of women who are disempowered, $H_{p}$, is $\frac{q}{n}$ , where$q$ is the number of women identified as disempowered and $n$ is the total number of women.

(v) Compute the intensity of disempowerment. To focus measurement on the situation of the disempowered, we censor the inadequacy scores.^[[2]](#footnote-3)^ The censored inadequacy score, $c_{i}(k)$, for individual $i$ is equal to the inadequacy score if the individual is disempowered (i.e., if $c_{i}>k$, then $c_{i}\left( k \right)= c_{i}$). The censored inadequacy score, $c_{i}(k)$, is equal to zero if the individual is empowered (i.e., if $c_{i}\leq k$, then $c_{i}\left( k \right)=0$). The intensity (or breadth) of disempowerment ($A_{p}$) is the average inadequacy score of disempowered women:

$$A_{p}=\frac{\sum_{i=1}^{n} c_{i}\left( k \right)}{q}.$$

(vi) Compute the index of disempowerment *M*_0_ and the 3DE. With $M_{0}$, the disempowerment headcount ratio is adjusted for the intensity of disempowerment. $M_{0}$ is calculated as the product of the disempowerment headcount ratio and the intensity of disempowerment, $M_{0}=H_{p}\times A_{p}$, or, more simply, as the average censored inadequacy score among women:

$$M_{0}=\frac{1}{n}\sum_{i=1}^{n} c_{i}\left( k \right).$$

The 3DE is easily obtained:

$3DE=1-M_{0}=1-(H_{p}\times A_{p})$.

Although based on *M*_0_, the 3DE also can be expressed equivalently as:

$3DE=H_{e}+(H_{p}\times A_{e})$,

where $H_{e}$ is the empowered headcount ratio, which equals (1– $H_{p}$), and $A_{e}$ is the average adequacy score of disempowered women, which equals (1 – $A_{p}$).

*M*_0_ has two properties that can be useful for understanding disempowerment and analyzing the effects of a project: dimensional breakdown and subgroup decomposition.

a) Dimensional breakdown

*M*_0_ can be decomposed into the contribution of each indicator. This can be useful for diagnostic purposes—understanding which indicators to target to achieve greater increases in empowerment—and reveals broad patterns of how people are disempowered. Continuing our focus on the inadequacies of the disempowered, we begin the decomposition by censoring the inadequacy status for each individual, replacing with zero the inadequacies of the empowered (as above, $g_{ij}\left( k \right)=g_{ij}$ if $c_{i}\geq k$ and $g_{ij}\left( k \right)=0$, otherwise). Then, we compute the censored inadequacy headcount ratios. The censored inadequacy headcount ratio of indicator $j$, denoted $h_{j}(k)$, is the proportion of the population that is both disempowered and simultaneously inadequate in that indicator. Formally:

$$h_{j}(k)=\frac{1}{n}\sum_{i=1}^{n} g_{ij}\left( k \right).$$

Thus, *M*_0_ can also be written as the weighted sum of the censored headcount ratios:

$$M_{0}=\sum_{j=1}^{d} w_{j}\times h_{j}(k).$$

The absolute contribution to disempowerment of indicator $j$ is $w_{j}\times h_{j}(k)$ and the relative contribution is $\frac{w_{j}\times h_{j}(k)}{M_{0}}$. Whenever the relative contribution to disempowerment of an indicator greatly exceeds its weight, this suggests that the disempowered are disproportionally more inadequate in this indicator compared to other indicators.

*b) Subgroup decomposition*

*M*_0_ also can be disaggregated by subgroups, such as treatment arms, depending on sample design and as long as the respective groups are mutually exclusive and exhaustive of the total sample (Alkire et al. 2015).^[[3]](#footnote-4)^ Disaggregating *M*_0_, and more generally pro-WEAI, by subgroup requires that the underlying data are statistically representative of the subgroup. The subgroup decomposition is calculated as:

$$M_{0}=\sum_{l=1}^{m} \frac{n^{l}}{n}M_{0}^{l},$$

where $M_{0}^{l}$ denotes the *M*_0_ of group $l\in\left[ 1,m \right]$ and $\frac{n^{l}}{n}$ denotes the population share of that same group. Thus, the relative contribution to disempowerment of group $l$ is $\frac{\frac{n^{l}}{n}\times M_{0}^{l}}{M_{0}}$. As before, whenever the relative contribution to disempowerment of a group greatly exceeds its population share, this suggests that the group may bear a disproportionate share of disempowerment.

### Supplement Appendix A.2 Gender Parity Index (GPI)

The GPI focuses on the difference between the inadequacy scores of the eligible woman and her spouse within each household. In contrast to the 3DE, which focuses on women’s inadequacy scores and is based on the full sample of women, the GPI involves the calculation of inadequacy scores for men and women and is based on the sample of dual-adult households (i.e., comprised of at least one woman and one man). Although in most cases the two adults compared will be a woman and her spouse, this is not a requirement.

Similar to the 3DE, the GPI is defined in terms of empowerment. Its construction stems from the identification of households that lack gender parity. The steps to construct the GPI are described below.

(i) Censor the inadequacy scores for gender parity. The inadequacy scores of men or women who are empowered, i.e., whose inadequacy scores are less than or equal to the disempowerment cut-off $k$, are replaced by the value of $k$ (rather than zero as in the computation of the 3DE). The new censored inadequacy score, denoted as ${c^{'}}_{i}\left( k \right)$ to differentiate it from the 3DE, is defined as follows: ${c'}_{i}\left( k \right)= c_{i}$ if $c_{i}>k$, and ${c'}_{i}\left( k \right)=k$ if $c_{i}\leq k$.

(ii) Identify households lacking gender parity. A household lacks gender parity if the woman is disempowered and her new censored inadequacy score is higher than the new censored inadequacy score of her male counterpart. Formally, household $j$ lacks gender parity if ${{c'}_{j}\left( k \right)}^{W}>k$ and ${{c^{'}}_{j}\left( k \right)}^{W}>{{c^{'}}_{j}\left( k \right)}^{M}$, where ${{c'}_{j}\left( k \right)}^{W}$ and ${{c'}_{j}\left( k \right)}^{M}$ are the censored inadequacy scores of the eligible woman and spouse, respectively. Put differently, a household is identified as achieving gender parity if the woman is empowered or, if she is not empowered, her inadequacy score is equal or lower than that of the man in her household.

(iv) Compute the proportion of households lacking gender parity. The proportion of households where women lack gender parity relative to their male counterparts, ${(H}_{GPI})$ is $r/m$, where *r* is the number of households classified as lacking gender parity and *m* is the total number of dual-adult households in the sample.

(v) Compute the average empowerment gap. The empowerment gap captures the extent of the disparity between women’s and men’s inadequacy scores in households that lack gender parity. It is calculated as the average relative gap in the censored inadequacy scores between women and men living in households that lack gender parity:

$I_{GPI}=\frac{1}{r}\sum_{j=1}^{r} \frac{{{c'}_{j}\left( k \right)}^{W}-{{c'}_{j}\left( k \right)}^{M}}{1-{{c'}_{j}\left( k \right)}^{M}}$.

(vi) Computing the GPI. The GPI combines the two last figures: the percentage of women who lack gender parity and the average empowerment gap:^[[4]](#footnote-5)^

$$GPI=1-{(H}_{GPI}\times I_{GPI}).$$

Like the 3DE, the GPI is decomposable by subgroups.

# Supplement Appendix B: Pro-WEAI survey modules

MODULE G. WOMEN’S EMPOWERMENT IN AGRICULTURE INDEX – Pilot Pro-WEAI Version

| **Note to survey designers:** The information in module G1 can be captured in different ways; however, there must be a way to: (a) identify the proper individual within the household to be asked the survey, (b) link this individual from the module to the household roster, (c) code the outcome of the interview, especially if the individual is not available, to distinguish this from missing data, and (d) record who else in the household was present during the interview. This instrument must be adapted for country context including adding relevant examples and translations into local languages when appropriate.  ***Note to enumerators:*** *This questionnaire should be administered separately to the primary and secondary respondents identified in the household roster of the household level questionnaire. You should complete this coversheet for each individual identified in the “selection section” even if the individual is not available to be interviewed for reporting purposes. For some surveys (such as those focusing on nutrition outcomes), the female respondent may be the beneficiary woman or mother or primary caregiver of the index child (also the respondent for the pro-WEAI nutrition module). Please make sure that she is also the person interviewed for this questionnaire and that the male respondent is her spouse/partner (if applicable).*  Please double-check to ensure:  You have completed the roster section of the household questionnaire to identify the correct primary and/or secondary respondent(s);  You have noted the household ID and individual ID correctly for the person you are about to interview;  You have gained informed consent from the individual in the household questionnaire;  You have sought to interview the individual in private or where other members of the household cannot overhear or contribute answers.  Do not attempt to make responses between the primary and secondary respondents the same—it is okay for them to be different. |
| --- |

**MODULE G1. INDIVIDUAL IDENTIFICATION**

| **G1.01. Household Identification:** | \|  \|  \|  \|  \|  \|  \| \| --- \| --- \| --- \| --- \| --- \| --- \| | **G1.04 TYPE OF HOUSEHOLD** | Male and female adult…………………………………………………………1  Female adult only………………………………………………………………..2 |
| --- | --- | --- | --- | --- | --- | --- | --- | --- | --- |
| **G1.02. Name of respondent currently being interviewed (ID Code from roster in Section B Household Roster):** | \|  \|  \| \| --- \| --- \| | **G1.05. Outcome of interview:**  **CIRCLE ONE** | Completed……………………………………………………………………………1  HOUSEHOLD MEMBER TOO ILL TO RESPOND/COGNITIVELY IMPAIRED…2  RESPONDENT Not at home/temporarily unavailable……………….3  RESPONDENT Not at home/extended absence…………………………4  REFUSED…………………………………………………………………………….…5  Could not locate…………………………………………………………………6 |
| **Surname, OTHER NAME: ____________________________________________________** | |  |  |
| **G1.03. Sex of respondent:** | Male…………………………….1  FEMale…………………………2 | **G1.06. Ability to be interviewed alone:**  **CIRCLE ONE** | Alone…………………………………………………………………………………..1  With adult females present…………………………………………………2  With adult males present…………………………………………………….3  With adults of BOTH sex present………………………………………….4  With children present…………………………………………………………5  With adults OF BOTH sex and children present…………………….6 |

| **HOUSEHOLD IDENTIFICATION (IN DATA FILE, EACH SUB-MODULE (G2-G8) MUST BE LINKED WITH A HH AND RESPONDENT ID)** | | | | | | | | | **HOUSEHOLD ID** | |  |  | |  |  |  |  |
| --- | --- | --- | --- | --- | --- | --- | --- | --- | --- | --- | --- | --- | --- | --- | --- | --- | --- |
|  |  |  |  |  |  |  |  |  | **RESPONDENT ID** | | | | | | |  |  |
| **MODULE G2: ROLE IN HOUSEHOLD DECISION-MAKING AROUND PRODUCTION AND INCOME** | | | | | | | | | | | | | | | | | |
| Now I’d like to ask you some questions about your participation in certain types of work activities and on making decisions on various aspects of household life. | | Did you [NAME] participate in [ACTIVITY] in the past 12 months (that is, during the last [one/two] cropping seasons), from [PRESENT MONTH] last year to [PRESENT MONTH] this year? | When decisions are made regarding [ACTIVITY], who is it that normally takes the decision?  **ENTER UP TO THREE (3) MEMBER IDs**  **IF RESPONSE IS MEMBER ID (SELF) ONLY** 🡪 ***G2.05***  **OTHER CODES:**  NON-HH MEMBER...….94  NOT APPLICABLE….…98 🡪 ***NEXT ACTIVITY*** | | | How much input did you have in making decisions about [ACTIVITY]?  **USE CODE G2↓** | To what extent do you feel you can participate in decisions regarding [ACTIVITY] if you want(ed) to?  **CIRCLE ONE** | To what extent are you able to access information that you feel is important for making informed decisions regarding [ACTIVITY]?  **CIRCLE ONE** | | How much input did you have in decisions about how much of the outputs of [ACTIVITY] to keep for consumption at home rather than selling?  **USE CODE G2↓** | | | How much input did you have in decisions about how to use income generated from [ACTIVITY]?  **USE CODE G2↓** | | | | |
| **ACTIVITY** | | **G2.01** | **G2.02** | | | **G2.03** | **G2.04** | **G2.05** | | **G2.06** | | | **G2.07** | | | | |
|  |  |  | **ID #1** | **ID #2** | **ID #3** |  |  |  |  |  |  |  |  |  |  |  |  |
| **A** | Staple grain farming and processing of the harvest: grains that are grown primarily for food consumption (rice, maize, wheat, millet) | Yes…...1  No…….2 🡪 ***activity B*** |  |  |  |  | Not at all……….…1  Small extent……..2  Medium extent…...3  To a high extent...4 | Not at all……….…1  Small extent……..2  Medium extent…...3  To a high extent...4 | |  | | |  | | | | |
| **B** | Horticultural (gardens) or high value crop farming and processing of the harvest | Yes…...1  No…….2 🡪 ***activity C*** |  |  |  |  | Not at all……….…1  Small extent……..2  Medium extent…...3  To a high extent...4 | Not at all……….…1  Small extent……..2  Medium extent…...3  To a high extent...4 | |  | | |  | | | | |
| **C** | Large livestock raising (cattle, buffaloes) and processing of milk and/or meat | Yes…...1  No…….2 🡪 ***activity D*** |  |  |  |  | Not at all……….…1  Small extent……..2  Medium extent…...3  To a high extent...4 | Not at all……….…1  Small extent……..2  Medium extent…...3  To a high extent...4 | |  | | |  | | | | |
| **D** | Small livestock raising (sheep, goats, pigs) and processing of milk and/or meat | Yes…...1  No…….2 🡪 ***activity E*** |  |  |  |  | Not at all……….…1  Small extent……..2  Medium extent…...3  To a high extent...4 | Not at all……….…1  Small extent……..2  Medium extent…...3  To a high extent...4 | |  | | |  | | | | |
| **E** | Poultry and other small animals raising (chickens, ducks, turkeys) and processing of eggs and/or meat | Yes…...1  No…….2 🡪 ***activity F*** |  |  |  |  | Not at all……….…1  Small extent……..2  Medium extent…...3  To a high extent...4 | Not at all……….…1  Small extent……..2  Medium extent…...3  To a high extent...4 | |  | | |  | | | | |

| **CODE G2** |
| --- |
| LITTLE TO NO INPUT IN DECISIONS 1  Input into some decisions 2  Input into most OR ALL decisions 3  NOT APPICABLE / No decision made 98 |

|  | | Did you [NAME] participate in [ACTIVITY] in the past 12 months (that is, during the last [one/two] cropping seasons), from [PRESENT MONTH] last year to [PRESENT MONTH] this year? | When decisions are made regarding [ACTIVITY], who is it that normally takes the decision?  **ENTER UP TO THREE (3) MEMBER IDs**  **IF RESPONSE IS MEMBER ID (SELF) ONLY** 🡪 ***G2.05***  **OTHER CODES:**  NON-HH MEMBER...….94  NOT APPLICABLE….…98 🡪 ***NEXT ACTIVITY*** | | | | How much input did you have in making decisions about [ACTIVITY]?  **USE CODE G2↓** | To what extent do you feel you can participate in decisions regarding [ACTIVITY] if you want(ed) to?  **CIRCLE ONE** | To what extent are you able to access information that you feel is important for making informed decisions regarding [ACTIVITY]?  **CIRCLE ONE** | How much input did you have in decisions about how much of the outputs of [ACTIVITY] to keep for consumption at home rather than selling?  **USE CODE G2↓** | How much input did you have in decisions about how to use income generated from [ACTIVITY]?  **USE CODE G2↓** |
| --- | --- | --- | --- | --- | --- | --- | --- | --- | --- | --- | --- |
| **ACTIVITY** | | **G2.01** | **G2.02** | | | | **G2.03** | **G2.04** | **G2.05** | **G2.06** | **G2.07** |
|  |  |  | **ID #1** | | **ID #2** | **ID #3** |  |  |  |  |  |
| **F** | Fishpond culture | Yes…...1  No…….2 🡪 ***activity G*** |  | |  |  |  | Not at all……….…1  Small extent……..2  Medium extent…...3  To a high extent...4 | Not at all……….…1  Small extent……..2  Medium extent…...3  To a high extent...4 |  |  |
| **G** | Non-farm economic activities (running a small business, self-employment, buy-and-sell) | Yes…...1  No…….2 🡪 ***activity H*** |  | |  |  |  | Not at all……….…1  Small extent……..2  Medium extent…...3  To a high extent...4 | Not at all……….…1  Small extent……..2  Medium extent…...3  To a high extent...4 |  |  |
| **H** | Wage and salary employment (work that is paid for in cash or in-kind, including both agriculture and other wage work) | Yes…...1  No…….2 🡪 ***activity I*** | |  |  |  |  | Not at all……….…1  Small extent……..2  Medium extent…...3  To a high extent...4 | Not at all……….…1  Small extent……..2  Medium extent…...3  To a high extent...4 |  |  |
| **I** | Large, occasional household purchases (bicycles, land, transport vehicles) |  | |  |  |  |  | Not at all……….…1  Small extent……..2  Medium extent…...3  To a high extent...4 | Not at all……….…1  Small extent……..2  Medium extent…...3  To a high extent...4 |  |  |
| **J** | Routine household purchases (food for daily consumption or other household needs) |  | |  |  |  |  | Not at all……….…1  Small extent……..2  Medium extent…...3  To a high extent...4 | Not at all……….…1  Small extent……..2  Medium extent…...3  To a high extent...4 |  |  |

| **CODE G2** |
| --- |
| LITTLE TO NO INPUT IN DECISIONS 1  Input into some decisions 2  Input into most OR ALL decisions 3  NOT APPLICABLE / NO DECISION MADE 98 |

|  | **HOUSEHOLD ID** |  |  |  |  |  |  |
| --- | --- | --- | --- | --- | --- | --- | --- |
|  | **RESPONDENT ID** | | | | |  |  |

**MODULE G3(A): ACCESS TO PRODUCTIVE CAPITAL**

| Now I’d like to ask you specifically about your household’s land. | | | | |
| --- | --- | --- | --- | --- |
| **QUESTION** | | **RESPONSE** | | |
| **G3.01.** Does anyone in your household currently own or cultivate land? | | YES……..1  NO………2 🡪 ***G3.06, ITEM A*** | | |
| **G3.02.** Who generally makes decisions about what to plant on this land? | **ENTER UP TO THREE (3) MEMBER IDs**  **OTHER CODES:**  NON-HH MEMBER……………………….94  NOT APPLICABLE………………………..98 | **ID #1** | **ID #2** | **ID #3** |
|  |  |  |  |  |
| **G3.03.** Do you [NAME] solely or jointly cultivate any land? | **CIRCLE ONE** | YES, SOLELY 1  YES, JOINTLY 2  YES, SOLELY AND JOINTLY 3  NO 4 | | |
| **G3.04.** Who generally makes decisions about what to plant on the land that you yourself cultivate? | **ENTER UP TO THREE (3) MEMBER IDs**  **OTHER CODES:**  NON-HH MEMBER……………………….94  NOT APPLICABLE………………………..98 | **ID #1** | **ID #2** | **ID #3** |
|  |  |  |  |  |
| **G3.05.** Do you own any of the land owned or cultivated by your household? | **CIRCLE ONE** | YES, SOLELY 1  YES, JOINTLY 2  YES, SOLELY AND JOINTLY 3  NO 4 | | |

| Now I’d like to ask you about a number of items that could be used to generate income. | | Does anyone in your household currently have any [ITEM]? | Do you [NAME] own any [ITEM]?  **CIRCLE ONE** |
| --- | --- | --- | --- |
| **ITEM** | | **G3.06** | **G3.07** |
| **A** | Large livestock (cattle, buffaloes) | YES……..1  NO………2 🡪 ***item B*** | YES, SOLELY 1  YES, JOINTLY 2  YES, SOLELY AND JOINTLY 3  NO 4 |
| **B** | Small livestock (sheep, goats, pigs) | YES……..1  NO………2 🡪 ***item C*** | YES, SOLELY 1  YES, JOINTLY 2  YES, SOLELY AND JOINTLY 3  NO 4 |
| **C** | Poultry and other small animals (chickens, ducks, turkeys) | YES……..1  NO………2 🡪 ***item D*** | YES, SOLELY 1  YES, JOINTLY 2  YES, SOLELY AND JOINTLY 3  NO 4 |
| **D** | Fish pond or fishing equipment | YES……..1  NO………2 🡪 ***item E*** | YES, SOLELY 1  YES, JOINTLY 2  YES, SOLELY AND JOINTLY 3  NO 4 |
| **E** | Non-mechanized farm equipment (hand tools, animal-drawn plough) | YES……..1  NO………2 🡪 ***item F*** | YES, SOLELY 1  YES, JOINTLY 2  YES, SOLELY AND JOINTLY 3  NO 4 |
| **F** | Mechanized farm equipment (tractor-plough, power tiller, treadle pump) | YES……..1  NO………2 🡪 ***item G*** | YES, SOLELY 1  YES, JOINTLY 2  YES, SOLELY AND JOINTLY 3  NO 4 |
| **G** | Non-farm business equipment (solar panels used for recharging, sewing machine, brewing equipment, fryers) | YES……..1  NO………2 🡪 ***item H*** | YES, SOLELY 1  YES, JOINTLY 2  YES, SOLELY AND JOINTLY 3  NO 4 |
| **H** | House or building | YES……..1  NO………2 🡪 ***item I*** | YES, SOLELY 1  YES, JOINTLY 2  YES, SOLELY AND JOINTLY 3  NO 4 |
| **I** | Large consumer durables (refrigerator, TV, sofa) | YES……..1  NO………2 🡪 ***item J*** | YES, SOLELY 1  YES, JOINTLY 2  YES, SOLELY AND JOINTLY 3  NO 4 |

|  | | Does anyone in your household currently own any [ITEM]? | Do you [NAME] own any [ITEM]?  **CIRCLE ONE** |
| --- | --- | --- | --- |
| **ITEM** | | **G3.06** | **G3.07** |
| **J** | Small consumer durables (radio, cookware) | YES……..1  NO………2 🡪 ***item K*** | YES, SOLELY 1  YES, JOINTLY 2  YES, SOLELY AND JOINTLY 3  NO 4 |
| **K** | Cell phone | YES……..1  NO………2 🡪 ***item L*** | YES, SOLELY 1  YES, JOINTLY 2  YES, SOLELY AND JOINTLY 3  NO 4 |
| **L** | Other land not used for agricultural purposes (pieces/plots, residential or commercial land) | YES……..1  NO………2 🡪 ***item M*** | YES, SOLELY 1  YES, JOINTLY 2  YES, SOLELY AND JOINTLY 3  NO 4 |
| **M** | Means of transportation (bicycle, motorcycle, car) | YES……..1  NO………2 🡪 ***MODULE G3(B)*** | YES, SOLELY 1  YES, JOINTLY 2  YES, SOLELY AND JOINTLY 3  NO 4 |

MODULE G3(B): ACCESS TO FINANCIAL SERVICES

| Next I’d like to ask about your household’s experience with borrowing money or other items (in-kind) in the past 12 months. | | Would you or anyone in your household be able to take a loan or borrow cash/in-kind from [SOURCE] if you wanted to? | Has anyone in your household taken any loans or borrowed cash/in-kind from [SOURCE] in the past 12 months?  **CIRCLE ONE** | Who made the decision to borrow from [SOURCE] most of the time?  **ENTER UP TO THREE (3) MEMBER IDs**  **OTHER CODES:**  NON-HH MEMBER...….94  NOT APPLICABLE….…98 | | | Who makes the decision about what to do with the money or item borrowed from [SOURCE] most of the time?  **ENTER UP TO THREE (3) MEMBER IDs**  **OTHER CODES:**  NON-HH MEMBER...….94  NOT APPLICABLE….…98 | | | Who is responsible for repaying the money or item borrowed from [SOURCE]?  **ENTER UP TO THREE (3) MEMBER IDs**  **OTHER CODES:**  NON-HH MEMBER...….94  NOT APPLICABLE….…98 | | |
| --- | --- | --- | --- | --- | --- | --- | --- | --- | --- | --- | --- | --- |
| **Lending sources** | | **G3.08** | **G3.09** | **G3.10** | | | **G3.11** | | | **G3.12** | | |
|  |  |  |  | **ID #1** | **ID #2** | **ID #3** | **ID #1** | **ID #2** | **ID #3** | **ID #1** | **ID #2** | **ID #3** |
| **A** | Non-governmental organization (NGO) | YES...…….1  NO………..2 🡪 ***SOURCE B***  MAYBE.….3 | YES, CASH 1  YES, IN-KIND 2  YES, CASH AND IN-KIND 3  NO 4 ***SOURCE B***  DON’T KNOW 97 |  |  |  |  |  |  |  |  |  |
| **B** | Formal lender (bank/financial institution) | YES...…….1  NO………..2 🡪 ***SOURCE C***  MAYBE.….3 | YES, CASH 1  YES, IN-KIND 2  YES, CASH AND IN-KIND 3  NO 4 ***SOURCE C*** DON’T KNOW 97 |  |  |  |  |  |  |  |  |  |
| **C** | Informal lender | YES...…….1  NO………..2 🡪 ***SOURCE D***  MAYBE.….3 | YES, CASH 1  YES, IN-KIND 2  YES, CASH AND IN-KIND 3  NO 4 ***SOURCE D*** DON’T KNOW 97 |  |  |  |  |  |  |  |  |  |
| **D** | Friends or relatives | YES...…….1  NO………..2 🡪 ***SOURCE E***  MAYBE.….3 | YES, CASH 1  YES, IN-KIND 2  YES, CASH AND IN-KIND 3  NO 4 ***SOURCE E*** DON’T KNOW 97 |  |  |  |  |  |  |  |  |  |
| **E** | Group based micro-finance or lending including VSLAs / SACCOs | YES...…….1  NO………..2 🡪 ***SOURCE F***  MAYBE.….3 | YES, CASH 1  YES, IN-KIND 2  YES, CASH AND IN-KIND 3  NO 4 ***SOURCE F*** DON’T KNOW 97 |  |  |  |  |  |  |  |  |  |
| **F** | Informal credit / savings groups (.e.g., merry-go-rounds, tontines, funeral societies, etc.) | YES...…….1  NO………..2 🡪 ***G3.13***  MAYBE.….3 | YES, CASH 1  YES, IN-KIND 2  YES, CASH AND IN-KIND 3  NO 4 ***G3.13***  DON’T KNOW 97 |  |  |  |  |  |  |  |  |  |

| **G3.13** | An account can be used to save money, to make or receive payments, or to receive wages or financial help. Do you, either by yourself or together with someone else, currently have an account at any of the following places: a bank or other formal institution (e.g., post office)? | YES 1  NO 2  DON’T KNOW 97 |
| --- | --- | --- |

|  | **HOUSEHOLD ID** |  |  |  |  |  |  |
| --- | --- | --- | --- | --- | --- | --- | --- |
|  | **RESPONDENT ID** | | | | |  |  |

**MODULE G4: TIME ALLOCATION**

**G4.01:** Please record a log of the activities for the individual in the last complete 24 hours (starting yesterday morning at 4 am, finishing 3:59 am of the current day). The time intervals are marked in 15 min intervals. mark one activity for each time period by ENTERING THE CORRESPONDING ACTIVITY CODE in the box.

**G4.02:** CHECK THE BOX BELOW IF THE RESPONDENT WAS CARING FOR CHILDREN WHILE PERFORMING EACH ACTIVITY.

| Now I’d like to ask you about how you spent your time during the past 24 hours. We’ll begin from yesterday morning, and continue through to this morning. This will be a detailed accounting. I’m interested in everything you did (i.e. resting, eating, personal care, work inside and outside the home, caring for children, cooking, shopping, socializing, etc.), even if it didn’t take you much time. I’m particularly interested in agricultural activities such as farming, gardening, and livestock raising whether in the field or on the homestead. I’m also interested in how much time you spent caring for children, especially if it happened while you did some other activity (e.g., collecting water while carrying a child or cooking while watching after a sleeping child). | | | | | | | | | | | | | | | | | | | | | | | | | | | | | | | | | | | | | | | | | | | | | | | | | | | | | | | | | | | | | | | | | | | | | | | | | | | | | | | | | | | | | |
| --- | --- | --- | --- | --- | --- | --- | --- | --- | --- | --- | --- | --- | --- | --- | --- | --- | --- | --- | --- | --- | --- | --- | --- | --- | --- | --- | --- | --- | --- | --- | --- | --- | --- | --- | --- | --- | --- | --- | --- | --- | --- | --- | --- | --- | --- | --- | --- | --- | --- | --- | --- | --- | --- | --- | --- | --- | --- | --- | --- | --- | --- | --- | --- | --- | --- | --- | --- | --- | --- | --- | --- | --- | --- | --- | --- | --- | --- | --- | --- | --- | --- | --- | --- | --- | --- |
|  | | **Night** | | | | | | | | | | | | | | | | | | | **Morning** | | | | | | | | **Day** | | | | | | | | | | | | | | | | | | | | | | | | | | | | | | | | | | | | | | | | | | | | | | | | | | | | | | | | |
|  |  | 4:00 | | | | | | | | | | 5:00 | | | | | | | | | 6:00 | | | | | | | | 7:00 | | | | | | | | | 8:00 | | | | | | | 9:00 | | | | | | | | | | 10:00 | | | | | | | 11:00 | | | | | 12:00 | | | | | 13:00 | | | | | 14:00 | | | | | 15:00 | | | |
| **G4.01** Activity **(WRITE ACTIVITY CODE)** | |  | |  | |  | |  | | | |  |  | |  | |  | | | |  |  |  | |  | | | |  | |  | |  | |  | | |  | |  | |  |  | |  | |  | |  | |  | | |  | | |  | |  |  | |  |  |  |  | |  |  |  |  | |  |  |  |  | |  |  |  |  | |  |  |  |  |
| **G4.02** Did you also care for children?  YES..…CHECK BOX  NO…LEAVE BLANK | YES CHECK BOX  NO LEAVE BLANK | □ | | □ | | □ | | □ | | | | □ | □ | | □ | | □ | | | | □ | □ | □ | | □ | | | | □ | | □ | | □ | | □ | | | □ | | □ | | □ | □ | | □ | | □ | | □ | | □ | | | □ | | | □ | | □ | □ | | □ | □ | □ | □ | | □ | □ | □ | □ | | □ | □ | □ | □ | | □ | □ | □ | □ | | □ | □ | □ | □ |
|  | | **Day** | | | | | | | | | | | | | | | | | **Evening** | | | | | | | | **Night** | | | | | | | | | | | | | | | | | | | | | | | | | | | | | | | | | | | | | | | | | | | | | | | | | | | | | | | | | | |
|  |  | 16:00 | | | | | | | | 17:00 | | | | | | | | | 18:00 | | | | | | | | 19:00 | | | | | | | | | 20:00 | | | | | | | | 21:00 | | | | | | | | | 22:00 | | | | | | | | 23:00 | | | | | 24:00 | | | | | 1:00 | | | | | 2:00 | | | | | 3:00 | | | | |
| **G4.01** Activity **(WRITE ACTIVITY CODE)** | | |  | |  | |  | |  | |  | | |  | |  | |  | |  | |  | |  | |  | |  | |  | |  | |  | | |  | |  | |  | |  | | |  | |  | |  | |  | | |  |  | |  | |  | |  |  |  |  | |  |  |  |  | |  |  |  |  | |  |  |  |  | |  |  |  |  |
| **G4.02** Did you also care for children? | YES CHECK BOX  NO LEAVE BLANK | | □ | | □ | | □ | | □ | | □ | | | □ | | □ | | □ | | □ | | □ | | □ | | □ | | □ | | □ | | □ | | □ | | | □ | | □ | | □ | | □ | | | □ | | □ | | □ | | □ | | | □ | □ | | □ | | □ | | □ | □ | □ | □ | | □ | □ | □ | □ | | □ | □ | □ | □ | | □ | □ | □ | □ | | □ | □ | □ | □ |

| **ACTIVITY CODES FOR G4.01** | | | |
| --- | --- | --- | --- |
| A………………Sleeping and resting  B.......................Eating and drinking  C……………...………Personal care  D………….School (incl. homework)  E……………..…Work as employed  F…………….…Own business work  G……………...Staple grain farming | H…………Horticultural (gardens) or high value crop farming  I………………..…..Large livestock raising (cattle, buffaloes)  J……………...….Small livestock raising (sheep, goats, pigs)  K...............................Poultry and other small animals raising  (chickens, ducks, turkeys)  L…………………………………………….…Fishpond culture  M……………………….Commuting (to/from work or school) | N………..Shopping / getting service (incl. health services)  O………………………..…..Weaving / sewing / textile care  P………………………………………………….…..Cooking  Q…..………..Domestic work (incl. fetching water and fuel)  R…………………………………………..Caring for children  S………………………..…..Caring for adults (sick, elderly)  T……………………..…..Traveling (not for work or school) | U………………………...Exercising  V……Social activities and hobbies  W…….…………Religious activities  X………………..….Other (specify) |

| **G4.03.** In the last 24 hours did you work (at home or outside of the home including chores or other domestic activities) less than usual, about the same as usual, or more than usual? | **FOR FEMALES ONLY:**  **DOES RESPONDENT HAVE A CHILD UNDER 5 YEARS OLD?**    YES...…….1 🡪 G4.04  NO………..2 🡪 MODULE G5 | **G4.04.** If you wanted to do something (livelihood-related, training-related, self-care) and could not take your child with you, is there someone who could care for your child in your absence?  YES...…….1 🡪 G4.05  NO………..2 🡪 MODULE G5 | **G4.05.** Who?  **ENTER UP TO THREE (3) MEMBER IDs**  **OTHER CODES:**  NON-HH MEMBER...….94  NOT APPLICABLE….…98 | **ID #1** | **ID #2** | **ID #3** |
| --- | --- | --- | --- | --- | --- | --- |
| LESS THAN USUAL...........................…….1  ABOUT THE SAME AS USUAL…………...2  MORE THAN USUAL……………………….3  **IF RESPONDENT IS MALE 🡪 MODULE G5** |  |  |  |  |  |  |

|  | **HOUSEHOLD ID** |  |  |  |  |  |  |
| --- | --- | --- | --- | --- | --- | --- | --- |
|  | **RESPONDENT ID** | | | | |  |  |

**MODULE G5: GROUP MEMBERSHIP**

| Now I’m going to ask you about groups in the community. These can be either formal or informal and customary groups. | | Is there a [GROUP] in your community? | | | Is this group composed of all male or female or mixed-sex members? | Are you an active member of this [GROUP]? | To what extent do you feel like you can influence decisions in this [GROUP]? | | To what extent does this [GROUP] influence life in the community beyond the group activities? | | |  |  |  |  |  |  |
| --- | --- | --- | --- | --- | --- | --- | --- | --- | --- | --- | --- | --- | --- | --- | --- | --- | --- |
| **Group Categories** | | **G5.01** | | | **G5.02** | **G5.03** | **G5.04** | | **G5.05** | | |  |  |  |  |  |  |
| **A** | Agricultural / livestock / fisheries producer’s group (including marketing groups) | YES 1  NO 2  DON’T KNOW 97 |  | ***GROUP B*** | ALL MALE……………………..1  ALL FEMALE………….……...2  MIXED SEX……………..…….3  DON’T KNOW……………….97 | YES……1  NO..……2 🡪 ***GROUP B*** | NOT AT ALL…………………...1  SMALL EXTENT………………2  MEDIUM EXTENT…………….3  HIGH EXTENT………………...4 | | NOT AT ALL…………………...1  SMALL EXTENT………………2  MEDIUM EXTENT…………….3  HIGH EXTENT………………...4 | | |  |  |  |  |  |  |
| **B** | Water users’ group | YES 1  NO 2  DON’T KNOW 97 |  | ***GROUP C*** | ALL MALE……………………..1  ALL FEMALE………….……...2  MIXED SEX……………..…….3  DON’T KNOW……………….97 | YES……1  NO..……2 🡪 ***GROUP C*** | NOT AT ALL…………………...1  SMALL EXTENT………………2  MEDIUM EXTENT…………….3  HIGH EXTENT………………...4 | | NOT AT ALL…………………...1  SMALL EXTENT………………2  MEDIUM EXTENT…………….3  HIGH EXTENT………………...4 | | |  |  |  |  |  |  |
| **C** | Forest users’ group | YES 1  NO 2  DON’T KNOW 97 |  | ***GROUP D*** | ALL MALE……………………..1  ALL FEMALE………….……...2  MIXED SEX……………..…….3  DON’T KNOW……………….97 | YES……1  NO..……2 🡪 ***GROUP D*** | NOT AT ALL…………………...1  SMALL EXTENT………………2  MEDIUM EXTENT…………….3  HIGH EXTENT………………...4 | | NOT AT ALL…………………...1  SMALL EXTENT………………2  MEDIUM EXTENT…………….3  HIGH EXTENT………………...4 | | |  |  |  |  |  |  |
| **D** | Credit or microfinance group (including SACCOs / merry-go-rounds / VSLAs) | YES 1  NO 2  DON’T KNOW 97 |  | ***GROUP E*** | ALL MALE……………………..1  ALL FEMALE………….……...2  MIXED SEX……………..…….3  DON’T KNOW……………….97 | YES……1  NO..……2 🡪 ***GROUP E*** | NOT AT ALL…………………...1  SMALL EXTENT………………2  MEDIUM EXTENT…………….3  HIGH EXTENT………………...4 | | NOT AT ALL…………………...1  SMALL EXTENT………………2  MEDIUM EXTENT…………….3  HIGH EXTENT………………...4 | | |  |  |  |  |  |  |
| **E** | Mutual help or insurance group (including burial societies) | YES 1  NO 2  DON’T KNOW 97 |  | ***GROUP F*** | ALL MALE……………………..1  ALL FEMALE………….……...2  MIXED SEX……………..…….3  DON’T KNOW……………….97 | YES……1  NO..……2 🡪 ***GROUP F*** | NOT AT ALL…………………...1  SMALL EXTENT………………2  MEDIUM EXTENT…………….3  HIGH EXTENT………………...4 | | NOT AT ALL…………………...1  SMALL EXTENT………………2  MEDIUM EXTENT…………….3  HIGH EXTENT………………...4 | | |  |  |  |  |  |  |
| **F** | Trade and business association group | YES 1  NO 2  DON’T KNOW 97 |  | ***GROUP G*** | ALL MALE……………………..1  ALL FEMALE………….……...2  MIXED SEX……………..…….3  DON’T KNOW……………….97 | YES……1  NO..……2 🡪 ***GROUP G*** | NOT AT ALL…………………...1  SMALL EXTENT………………2  MEDIUM EXTENT…………….3  HIGH EXTENT………………...4 | | NOT AT ALL…………………...1  SMALL EXTENT………………2  MEDIUM EXTENT…………….3  HIGH EXTENT………………...4 | | |  |  |  |  |  |  |
| **G** | Civic group (improving community) or charitable group (helping others) | YES 1  NO 2  DON’T KNOW 97 |  | ***GROUP H*** | ALL MALE……………………..1  ALL FEMALE………….……...2  MIXED SEX……………..…….3  DON’T KNOW……………….97 | YES……1  NO..……2 🡪 ***GROUP H*** | NOT AT ALL…………………...1  SMALL EXTENT………………2  MEDIUM EXTENT…………….3  HIGH EXTENT………………...4 | | NOT AT ALL…………………...1  SMALL EXTENT………………2  MEDIUM EXTENT…………….3  HIGH EXTENT………………...4 | | |  |  |  |  |  |  |
| **H** | Religious group | YES 1  NO 2  DON’T KNOW 97 |  | ***GROUP I*** | ALL MALE……………………..1  ALL FEMALE………….……...2  MIXED SEX……………..…….3  DON’T KNOW……………….97 | YES……1  NO..……2 🡪 ***GROUP I*** | NOT AT ALL…………………...1  SMALL EXTENT………………2  MEDIUM EXTENT…………….3  HIGH EXTENT………………...4 | | NOT AT ALL…………………...1  SMALL EXTENT………………2  MEDIUM EXTENT…………….3  HIGH EXTENT………………...4 | | |  |  |  |  |  |  |
| **I** | Other (specify): _______________________ | YES 1  NO 2  DON’T KNOW 97 |  | ***MODULE G6*** | ALL MALE……………………..1  ALL FEMALE………….……...2  MIXED SEX……………..…….3  DON’T KNOW……………….97 | YES……1  NO..……2 🡪 ***MODULE G6*** | NOT AT ALL…………………...1  SMALL EXTENT………………2  MEDIUM EXTENT…………….3  HIGH EXTENT………………...4 | | NOT AT ALL…………………...1  SMALL EXTENT………………2  MEDIUM EXTENT…………….3  HIGH EXTENT………………...4 | | |  |  |  |  |  |  |
|  | | | | | | | | | **HOUSEHOLD ID** | |  |  | |  |  |  |  |
|  |  |  |  |  |  |  |  |  | **RESPONDENT ID** | | | | | | |  |  |

**MODULE G6. PHYSICAL MOBILITY**

| **QUESTION** | **RESPONSE**  **FOR G6.01 - G6.06: USE CODE G6↓** |
| --- | --- |
| **G6.01** How often do you visit an urban center? |  |
| **G6.02** How often do you go to the market / haat / bazaar? |  |
| **G6.03** How often do you go to visit family or relatives? |  |
| **G6.04** How often do you go to visit a friend / neighbor’s house? |  |
| **G6.05** How often do you go to the hospital / clinic / doctor (seek health service)? |  |
| **G6.06** How often do you go to a public village gathering / community meeting / training for NGO or programs? |  |
| **G6.07.** In the last 12 months, how many times have you been away from home for one or more nights (in other words, sleeping somewhere else for the night)? |  |
| **G6.08.** In the last 12 months, have you been away from home for more than one month at a time? | YES…………………………………………………1  NO…………………………………………………..2  ***IF RESPONDENT IS MALE*** 🡪***MODULE G7*** |

| **CODE G6** |
| --- |
| EVERYDAY 1  EVERY WEEK AT LEAST ONCE 2  EVERY 2 WEEKS AT LEAST ONCE 3  EVERY MONTH AT LEAST ONCE 4  LESS THAN ONCE A MONTH 5  NEVER 6 |

**REMAINDER OF MODULE (G6.09-G6.08) SHOULD ONLY BE ASKED IF RESPONDENT IS FEMALE**

| Now I’d like to ask you some questions about different places you might visit. | | Who usually decides whether you can go to [PLACE]?  **ENTER UP TO THREE (3) MEMBER IDs**  **IF RESPONSE IS MEMBER ID (SELF) ONLY** 🡪 **NEXT PLACE**  **OTHER CODES:**  NON-HH MEMBER...….94  NOT APPLICABLE….…98 | | | Does your husband/partner or other household member object to you going alone to [PLACE]? | Under what circumstances would this person NOT object to your going to [PLACE] alone?  **CIRCLE ALL APPLICABLE** | Do these objections prevent you from going alone to [PLACE]? |
| --- | --- | --- | --- | --- | --- | --- | --- |
| **PLACE** | | **G6.09** | | | **G6.10** | **G6.11** | **G6.12** |
|  |  | **ID #1** | **ID #2** | **ID #3** |  |  |  |
| **A** | Urban center |  |  |  | YES……1  NO..……2 🡪 ***PLACE B*** | IF I HAVE COMPANY (RELATIVES, CHILDREN)………………………..….1  IF I CAN ARRANGE MY OWN EXPENSES (FOR TRANSPORT)………....2  IF I FOLLOW PURDAH / DRESS ACCEPTABLY…………………………...3  OTHER (SPECIFY)………………………………………………………………4  UNDER NO CIRCUMSTANCES WOULD I BE ALLOWED TO GO………..5 🡪 ***PLACE B*** | YES……1  NO..……2 |
| **B** | Market / haat / bazaar |  |  |  | YES……1  NO..……2 🡪 ***PLACE C*** | IF I HAVE COMPANY (RELATIVES, CHILDREN)………………………..….1  IF I CAN ARRANGE MY OWN EXPENSES (FOR TRANSPORT)………....2  IF I FOLLOW PURDAH / DRESS ACCEPTABLY…………………………...3  OTHER (SPECIFY)………………………………………………………………4  UNDER NO CIRCUMSTANCES WOULD I BE ALLOWED TO GO………..5 🡪 ***PLACE C*** | YES……1  NO..……2 |
| **C** | Visit family or relatives |  |  |  | YES……1  NO..……2 🡪 ***PLACE D*** | IF I HAVE COMPANY (RELATIVES, CHILDREN)………………………..….1  IF I CAN ARRANGE MY OWN EXPENSES (FOR TRANSPORT)………....2  IF I FOLLOW PURDAH / DRESS ACCEPTABLY…………………………...3  OTHER (SPECIFY)………………………………………………………………4  UNDER NO CIRCUMSTANCES WOULD I BE ALLOWED TO GO………..5 🡪 ***PLACE D*** | YES……1  NO..……2 |
| **D** | Visit a friend / neighbor’s house |  |  |  | YES……1  NO..……2 🡪 ***PLACE E*** | IF I HAVE COMPANY (RELATIVES, CHILDREN)………………………..….1  IF I CAN ARRANGE MY OWN EXPENSES (FOR TRANSPORT)………....2  IF I FOLLOW PURDAH / DRESS ACCEPTABLY…………………………...3  OTHER (SPECIFY)………………………………………………………………4  UNDER NO CIRCUMSTANCES WOULD I BE ALLOWED TO GO………..5 🡪 ***PLACE E*** | YES……1  NO..……2 |
| **E** | Hospital / clinic / doctor (seek health service) |  |  |  | YES……1  NO..……2 🡪 ***PLACE F*** | IF I HAVE COMPANY (RELATIVES, CHILDREN)………………………..….1  IF I CAN ARRANGE MY OWN EXPENSES (FOR TRANSPORT)………....2  IF I FOLLOW PURDAH / DRESS ACCEPTABLY…………………………...3  OTHER (SPECIFY)………………………………………………………………4  UNDER NO CIRCUMSTANCES WOULD I BE ALLOWED TO GO………..5 🡪 ***PLACE F*** | YES……1  NO..……2 |

|  | | Who usually decides whether you can go to [PLACE]?  **ENTER UP TO THREE (3) MEMBER IDs**  **IF RESPONSE IS MEMBER ID (SELF) ONLY** 🡪 **NEXT PLACE**  **OTHER CODES:**  NON-HH MEMBER...….94  NOT APPLICABLE….…98 | | | Does your husband/partner or other household member object to you going alone to [PLACE]? | Under what circumstances would this person NOT object to your going to [PLACE] alone?  **CIRCLE ALL APPLICABLE** | Do these objections prevent you from going alone to [PLACE]? |
| --- | --- | --- | --- | --- | --- | --- | --- |
| **PLACE** | | **G6.09** | | | **G6.10** | **G6.11** | **G6.12** |
|  |  | **ID #1** | **ID #2** | **ID #3** |  |  |  |
| **F** | Temple / church / mosque |  |  |  | YES……1  NO..……2 🡪 ***PLACE G*** | IF I HAVE COMPANY (RELATIVES, CHILDREN)………………………..….1  IF I CAN ARRANGE MY OWN EXPENSES (FOR TRANSPORT)………....2  IF I FOLLOW PURDAH / DRESS ACCEPTABLY…………………………...3  OTHER (SPECIFY)………………………………………………………………4  UNDER NO CIRCUMSTANCES WOULD I BE ALLOWED TO GO………..5 🡪 ***PLACE G*** | YES……1  NO..……2 |
| **G** | Public village gathering or community meeting |  |  |  | YES……1  NO..……2 🡪 ***PLACE H*** | IF I HAVE COMPANY (RELATIVES, CHILDREN)………………………..….1  IF I CAN ARRANGE MY OWN EXPENSES (FOR TRANSPORT)………....2  IF I FOLLOW PURDAH / DRESS ACCEPTABLY…………………………...3  OTHER (SPECIFY)………………………………………………………………4  UNDER NO CIRCUMSTANCES WOULD I BE ALLOWED TO GO………..5 🡪 ***PLACE H*** | YES……1  NO..……2 |
| **H** | Training for NGO / programs |  |  |  | YES……1  NO..……2 🡪 ***PLACE I*** | IF I HAVE COMPANY (RELATIVES, CHILDREN)………………………..….1  IF I CAN ARRANGE MY OWN EXPENSES (FOR TRANSPORT)………....2  IF I FOLLOW PURDAH / DRESS ACCEPTABLY…………………………...3  OTHER (SPECIFY)………………………………………………………………4  UNDER NO CIRCUMSTANCES WOULD I BE ALLOWED TO GO………..5 🡪 ***PLACE I*** | YES……1  NO..……2 |
| **I** | Outside your community or village |  |  |  | YES……1  NO..……2 🡪 ***MODULE G7*** | IF I HAVE COMPANY (RELATIVES, CHILDREN)………………………..….1  IF I CAN ARRANGE MY OWN EXPENSES (FOR TRANSPORT)………....2  IF I FOLLOW PURDAH / DRESS ACCEPTABLY…………………………...3  OTHER (SPECIFY)………………………………………………………………4  UNDER NO CIRCUMSTANCES WOULD I BE ALLOWED TO GO………..5 🡪 ***MODULE G7*** | YES……1  NO..……2 |

|  | **HOUSEHOLD ID** |  |  |  |  |  |  |
| --- | --- | --- | --- | --- | --- | --- | --- |
|  | **RESPONDENT ID** | | | | |  |  |

**MODULE G7: INTRAHOUSEHOLD RELATIONSHIPS**

| Now I’d like to ask you some questions about how you feel about some of other people in your household or family group and how you think they feel about you.  **ENTER MEMBER ID FOR EACH RELATION**  **OTHER CODES:**  NON-HH MEMBER...….94 | | | Do you [NAME] respect your [RELATION]? | Does your [RELATION] respect you? | Do you trust your [RELATION] to do things that are in your best interest? | When you disagree with your [RELATION], do you feel comfortable telling him/her that you disagree? | **IS [RELATION] THE OTHER RESPONDENT WITHIN THIS HOUSEHOLD?** | Is there a co-wife within your household? |
| --- | --- | --- | --- | --- | --- | --- | --- | --- |
| **RELATION** | | | **G7.02** | **G7.03** | **G7.04** | **G7.05** | **G7.06** | **G7.07** |
| **A** | Husband / wife | **ID #** | MOST OF THE TIME...........1  SOMETIMES………………..2  RARELY……………………..3  NEVER………………………4 | MOST OF THE TIME...........1  SOMETIMES………………..2  RARELY……………………..3  NEVER………………………4 | MOST OF THE TIME...........1  SOMETIMES………………..2  RARELY……………………..3  NEVER………………………4 | MOST OF THE TIME...........1  SOMETIMES………………..2  RARELY……………………..3  NEVER………………………4 | YES……1 🡪 ***RELATION C***  NO..……2 |  |
|  |  |  |  |  |  |  |  |  |
| **B** | Other respondent within the household | **ID #** | MOST OF THE TIME...........1  SOMETIMES………………..2  RARELY……………………..3  NEVER………………………4 | MOST OF THE TIME...........1  SOMETIMES………………..2  RARELY……………………..3  NEVER………………………4 | MOST OF THE TIME...........1  SOMETIMES………………..2  RARELY……………………..3  NEVER………………………4 | MOST OF THE TIME...........1  SOMETIMES………………..2  RARELY……………………..3  NEVER………………………4 |  |  |
|  |  |  |  |  |  |  |  |  |
| **C** | **IF RESPONDENT IS MALE:**  Father (or adapt this category to capture other important relationship)    **IF RESPONDENT IS FEMALE:** Mother-in-law | **ID #** | MOST OF THE TIME...........1  SOMETIMES………………..2  RARELY……………………..3  NEVER………………………4 | MOST OF THE TIME...........1  SOMETIMES………………..2  RARELY……………………..3  NEVER………………………4 | MOST OF THE TIME...........1  SOMETIMES………………..2  RARELY……………………..3  NEVER………………………4 | MOST OF THE TIME...........1  SOMETIMES………………..2  RARELY……………………..3  NEVER……………………….4***IF RESPONDENT IS***  ***MALE*** 🡪 ***MODULE G8(A)*** |  | YES……1  NO..……2 🡪 ***MODULE G8(A)*** |
|  |  |  |  |  |  |  |  |  |
| **D** | Most senior co-wife (the person who was in the household just before you, or, if you are the senior wife, the one who married into the household after you) | **ID #** | MOST OF THE TIME...........1  SOMETIMES………………..2  RARELY……………………..3  NEVER……………………….4 | MOST OF THE TIME...........1  SOMETIMES………………..2  RARELY……………………..3  NEVER……………………….4 | MOST OF THE TIME...........1  SOMETIMES………………..2  RARELY……………………..3  NEVER……………………….4 | MOST OF THE TIME...........1  SOMETIMES………………..2  RARELY……………………..3  NEVER……………………….4 |  |  |
|  |  |  |  |  |  |  |  |  |

|  | **HOUSEHOLD ID** |  |  |  |  |  |  |
| --- | --- | --- | --- | --- | --- | --- | --- |
|  | **RESPONDENT ID** | | | | |  |  |

**MODULE G8(A): AUTONOMY IN DECISION-MAKING**

| Now I am going to read you some stories about different farmers and their situations regarding different agricultural activities. This question format is different from the rest so take your time in answering. For each I will then ask you how much you are like or not like each of these people. We would like to know if you are completely different from them, similar to them, or somewhere in between. There are no right or wrong answers to these questions.  **READ ALOUD EACH STORY, SUBSEQUENT QUESTIONs, AND RESPONSE CODES. NAMES SHOULD BE ADOPTED TO LOCAL CONTEXT AND BE MALE/FEMALE DEPENDING ON THE SEX OF THE RESPONDENT. THE ORDER OF TOPICS A-D SHOULD BE RANDOMIZED, AND WITHIN EACH TOPIC, THE ORDER OF STORIES 1-4 SHOULD BE RANDOMIZED.** | | | Are you like this person?  **CIRCLE ONE** | Are you completely the same or somewhat the same?  **CIRCLE ONE** | Are you completely different or somewhat different?  **CIRCLE ONE** |
| --- | --- | --- | --- | --- | --- |
| **STORY** | | | **G8.01** | **G8.02** | **G8.03** |
| The types of crops to grow or raise for consumption and sale in market | **A1** | *“[PERSON’S NAME] cannot grow other types of crops here for consumption and sale in market. Beans, sweet potato and maize are the only crops that grow here.”* | YES...1  NO.....2 🡪 ***G8.03*** | COMPLETELY THE SAME….1 🡪 ***A2***  SOMEWHAT THE SAME…....2 🡪 ***A2*** | COMPLETELY DIFFERENT....1  SOMEWHAT DIFFERENT.......2 |
|  | **A2** | *“[PERSON’S NAME] is a farmer and grows beans, sweet potato, and maize because her spouse, or another person or group in her community tells her she must grow these crops. She does what they tell her to do.”* | YES...1  NO.....2 🡪 ***G8.03*** | COMPLETELY THE SAME….1 🡪 ***A3***  SOMEWHAT THE SAME…....2 🡪 ***A3*** | COMPLETELY DIFFERENT....1  SOMEWHAT DIFFERENT.......2 |
|  | **A3** | *“[PERSON’S NAME] grows the crops for agricultural production that her family or community expect. She wants them to approve of her as a good farmer.”* | YES...1  NO.....2 🡪 ***G8.03*** | COMPLETELY THE SAME….1 🡪 ***A4***  SOMEWHAT THE SAME…....2 🡪 ***A4*** | COMPLETELY DIFFERENT....1  SOMEWHAT DIFFERENT.......2 |
|  | **A4** | *“[PERSON’S NAME] chooses the crops that she personally wants to grow for consumption and sale in market and thinks are best for herself and her family. She values growing these crops. If she changed her mind, she could act differently.”* | YES...1  NO.....2 🡪 ***G8.03*** | COMPLETELY THE SAME….1 🡪 ***B1***  SOMEWHAT THE SAME…....2 🡪 ***B1*** | COMPLETELY DIFFERENT....1  SOMEWHAT DIFFERENT.......2 |
| Livestock raising | **B1** | *“[PERSON’S NAME] cannot raise any livestock other than what she has. These are all that do well here.”* | YES...1  NO.....2 🡪 ***G8.03*** | COMPLETELY THE SAME….1 🡪 ***B2***  SOMEWHAT THE SAME…....2 🡪 ***B2*** | COMPLETELY DIFFERENT....1  SOMEWHAT DIFFERENT.......2 |
|  | **B2** | *“[PERSON’S NAME] raises the types of livestock she does because her spouse, or another person or group in her community tell her she must use these breeds. She does what they tell her to do.”* | YES...1  NO.....2 🡪 ***G8.03*** | COMPLETELY THE SAME….1 🡪 ***B3***  SOMEWHAT THE SAME…....2 🡪 ***B3*** | COMPLETELY DIFFERENT....1  SOMEWHAT DIFFERENT.......2 |
|  | **B3** | *“[PERSON’S NAME] raises the kinds of livestock that her family or community expect. She wants them to approve of her as a good livestock raiser.”* | YES...1  NO.....2 🡪 ***G8.03*** | COMPLETELY THE SAME….1 🡪 ***B4***  SOMEWHAT THE SAME…....2 🡪 ***B4*** | COMPLETELY DIFFERENT....1  SOMEWHAT DIFFERENT.......2 |
|  | **B4** | *“[PERSON’S NAME] chooses the types of livestock that she personally wants to raise and thinks are good for herself and her family. She values raising these types. If she changed her mind, she could act differently.”* | YES...1  NO.....2 🡪 ***G8.03*** | COMPLETELY THE SAME….1 🡪 ***C1***  SOMEWHAT THE SAME…....2 🡪 ***C1*** | COMPLETELY DIFFERENT....1  SOMEWHAT DIFFERENT.......2 |

| **READ ALOUD EACH STORY, SUBSEQUENT QUESTIONs, AND RESPONSE CODES. NAMES SHOULD BE ADOPTED TO LOCAL CONTEXT AND BE MALE/FEMALE DEPENDING ON THE SEX OF THE RESPONDENT.** | | | Are you like this person?  **CIRCLE ONE** | Are you completely the same or somewhat the same?  **CIRCLE ONE** | Are you completely different or somewhat different?  **CIRCLE ONE** |
| --- | --- | --- | --- | --- | --- |
| **STORY** | | | **G8.01** | **G8.02** | **G8.03** |
| Taking crops or livestock (incl. eggs or milk) to the market (or not) | **C1** | *“There is no alternative to how much or how little of her crops or livestock [PERSON’S NAME] can take to the market. She is taking the only possible amount.”* | YES...1  NO.....2 🡪 ***G8.03*** | COMPLETELY THE SAME….1 🡪 ***C2***  SOMEWHAT THE SAME…....2 🡪 ***C2*** | COMPLETELY DIFFERENT....1  SOMEWHAT DIFFERENT.......2 |
|  | **C2** | *“[PERSON’S NAME] takes crops and livestock to the market because her spouse, or another person or group in her community tell her she must sell them there. She does what they tell her to do.”* | YES...1  NO.....2 🡪 ***G8.03*** | COMPLETELY THE SAME….1 🡪 ***C3***  SOMEWHAT THE SAME…....2 🡪 ***C3*** | COMPLETELY DIFFERENT....1  SOMEWHAT DIFFERENT.......2 |
|  | **C3** | *“[PERSON’S NAME] takes the crops and livestock to the market that her family or community expect. She wants them to approve of her.”* | YES...1  NO.....2 🡪 ***G8.03*** | COMPLETELY THE SAME….1 🡪 ***C4***  SOMEWHAT THE SAME…....2 🡪 ***C4*** | COMPLETELY DIFFERENT....1  SOMEWHAT DIFFERENT.......2 |
|  | **C4** | *“[PERSON’S NAME] chooses to take the crops and livestock to market that she personally wants to sell there, and thinks is best for herself and her family. She values this approach to sales. If she changed her mind, she could act differently.”* | YES...1  NO.....2 🡪 ***G8.03*** | COMPLETELY THE SAME….1 🡪 ***D1***  SOMEWHAT THE SAME…....2 🡪 ***D1*** | COMPLETELY DIFFERENT....1  SOMEWHAT DIFFERENT.......2 |
| How to use income generated from agricultural and non-agricultural activities | **D1** | *“There is no alternative to how [PERSON’S NAME] uses her income. How she uses her income is determined by necessity.”* | YES...1  NO.....2 🡪 ***G8.03*** | COMPLETELY THE SAME….1 🡪 ***D2***  SOMEWHAT THE SAME…....2 🡪 ***D2*** | COMPLETELY DIFFERENT....1  SOMEWHAT DIFFERENT.......2 |
|  | **D2** | *“[PERSON’S NAME] uses her income how her spouse, or another person or group in her community tell her she must use it there. She does what they tell her to do.”* | YES...1  NO.....2 🡪 ***G8.03*** | COMPLETELY THE SAME….1 🡪 ***D3***  SOMEWHAT THE SAME…....2 🡪 ***D3*** | COMPLETELY DIFFERENT....1  SOMEWHAT DIFFERENT.......2 |
|  | **D3** | *“[PERSON’S NAME] uses her income in the way that her family or community expect. She wants them to approve of her.”* | YES...1  NO.....2 🡪 ***G8.03*** | COMPLETELY THE SAME….1 🡪 ***D4***  SOMEWHAT THE SAME…....2 🡪 ***D4*** | COMPLETELY DIFFERENT....1  SOMEWHAT DIFFERENT.......2 |
|  | **D4** | *“[PERSON’S NAME] chooses to use her income how she personally wants to, and thinks is best for herself and her family. She values using her income in this way. If she changed her mind, she could act differently.”* | YES...1  NO.....2 🡪 ***G8.03*** | COMPLETELY THE SAME...1🡪***G8.04***  SOMEWHAT THE SAME….2 🡪***G8.04*** | COMPLETELY DIFFERENT....1  SOMEWHAT DIFFERENT.......2 |

**MODULE G8(B): NEW GENERAL SELF-EFFICACY SCALE**

| Now I’m going to ask you some questions about different feelings you might have. Please listen to each of the following statements. Think about how each statement relates to your life, and then tell me how much you agree or disagree with the statement on a scale of 1 to 5, where 1 means you “strongly disagree” and 5 means you “strongly agree.” (**Note: Randomize order of statements**) | | |
| --- | --- | --- |
| **Statements** | | **G8.04** |
| **A** | I will be able to achieve most of the goals that I have set for myself. | STRONGLY DISAGREE 1  DISAGREE 2  NEITHER AGREE NOR DISAGREE 3  AGREE 4  STRONGLY AGREE 5 |
| **B** | When facing difficult tasks, I am certain that I will accomplish them. | STRONGLY DISAGREE 1  DISAGREE 2  NEITHER AGREE NOR DISAGREE 3  AGREE 4  STRONGLY AGREE 5 |
| **C** | In general, I think that I can obtain outcomes that are important to me. | STRONGLY DISAGREE 1  DISAGREE 2  NEITHER AGREE NOR DISAGREE 3  AGREE 4  STRONGLY AGREE 5 |
| **D** | I believe I can succeed at most any endeavor to which I set my mind | STRONGLY DISAGREE 1  DISAGREE 2  NEITHER AGREE NOR DISAGREE 3  AGREE 4  STRONGLY AGREE 5 |
| **E** | I will be able to successfully overcome many challenges. | STRONGLY DISAGREE 1  DISAGREE 2  NEITHER AGREE NOR DISAGREE 3  AGREE 4  STRONGLY AGREE 5 |
| **F** | I am confident that I can perform effectively on many different tasks. | STRONGLY DISAGREE 1  DISAGREE 2  NEITHER AGREE NOR DISAGREE 3  AGREE 4  STRONGLY AGREE 5 |
| **G** | Compared to other people, I can do most tasks very well. | STRONGLY DISAGREE 1  DISAGREE 2  NEITHER AGREE NOR DISAGREE 3  AGREE 4  STRONGLY AGREE 5 |
| **H** | Even when things are tough, I can perform quite well. | STRONGLY DISAGREE 1  DISAGREE 2  NEITHER AGREE NOR DISAGREE 3  AGREE 4  STRONGLY AGREE 5 |

**MODULE G8(C): LIFE SATISFACTION**

| The following questions ask how satisfied you feel with your life as a whole, on a scale from 1 to 5, where 1 means you feel “very dissatisfied” and 5 means you feel “very satisfied.” | | |
| --- | --- | --- |
|  | **Statements** | **G8.05** |
| **A** | Overall, how satisfied are you with life as a whole these days? | VERY DISSATISFIED 1  DISSATISFIED 2  NEITHER SATISFIED NOR DISSATISFIED 3  SATISFIED 4  VERY SATISFIED 5 |
| **B** | Overall, how satisfied with your life were you 5 years ago? | VERY DISSATISFIED 1  DISSATISFIED 2  NEITHER SATISFIED NOR DISSATISFIED 3  SATISFIED 4  VERY SATISFIED 5 |
| **C** | As your best guess, overall how satisfied with your life do you expect to feel 5 years from today? | VERY DISSATISFIED 1  DISSATISFIED 2  NEITHER SATISFIED NOR DISSATISFIED 3  SATISFIED 4  VERY SATISFIED 5 |

| **HOUSEHOLD ID** |  |  |  |  |  |  |
| --- | --- | --- | --- | --- | --- | --- |
| **RESPONDENT ID** | | | | |  |  |

**MODULE G9. Attitudes about Domestic Violence**

| Now I would like to ask about your opinion on the following issues. Please keep in mind that I am not asking about your personal experience or whether the following scenarios have happened to you. I would only like to know whether you think the following issues are acceptable. | | In your opinion, is a husband justified in hitting or beating his wife in the following situations? |
| --- | --- | --- |
| **SITUATION** | | **G9.01** |
| **A** | If she goes out without telling him? | YES 1  NO 2  DON’T KNOW 97 |
| **B** | If she neglects the children? | YES 1  NO 2  DON’T KNOW 97 |
| **C** | If she argues with him? | YES 1  NO 2  DON’T KNOW 97 |
| **D** | If she refuses to have sex with him? | YES 1  NO 2  DON’T KNOW 97 |
| **E** | If she burns the food? | YES 1  NO 2  DON’T KNOW 97 |

1. In pro-WEAI, as in WEAI, we define the disempowerment cut-off as strict $(c_{i}>k)$, rather than weak $(c_{i}\geq k)$, as in Alkire and Foster (2011). [↑](#footnote-ref-2)
2. Alternatively, we could choose not to censor the inadequacy scores. While ostensibly simpler, this approach would not allow for distinguishing between areas of disempowerment common among disempowered individuals and areas of disempowerment common among empowered individuals. [↑](#footnote-ref-3)
3. Before decomposing by subgroups, it is ideal to test for (and confirm) measurement invariance by subgroups. Confirming measurement invariance allows us to assume that the same trait is being measured in both subgroups. For subgroups in which no population differences are expected, such as a randomly assigned treatment arm, this is not necessary. [↑](#footnote-ref-4)
4. The GPI is equivalent to one minus a “poverty gap” or $P_{1}$ measure of the Foster- Greer-Thorbecke (1984) family of poverty measures. [↑](#footnote-ref-5)
