# Supplementary material for: Designing for empowerment impact in agricultural development projects: Experimental evidence from the Agriculture, Nutrition, and Gender Linkages (ANGeL) project in Bangladesh
Source: World Dev. 2021 Oct;146:105622. doi: 10.1016/j.worlddev.2021.105622 (PMC8350314; doi:10.1016/j.worlddev.2021.105622)
Supplement: Supplementary Data 2 [file mmc2.docx]

Supplement 2. List of questionnaire modules administered to male and female respondents at baseline and endline

Male questionnaire

|  | Baseline | Endline | Included in female questionnaire? |
| --- | --- | --- | --- |
| Household composition and education (roster) | yes | yes | no |
| Employment | yes | yes | no |
| Assets owned by household (including owner IDs) | yes | yes | no |
| Savings | yes | yes | no |
| Loans | yes | yes | no |
| Land and water bodies owned and operated | yes | yes | no |
| Agriculture (plots, irrigation and harvest, input use, cost of renting equipment, labor use, fertilizer, seed, and pesticides, production, food grain stock) | yes | yes | no |
| Agricultural extension | yes | yes | no |
| Livestock and poultry | yes | yes | no |
| Fish culture | yes | yes | no |
| Marketing | yes | yes | no |
| Non-agricultural enterprises | yes | yes | no |
| Nonfood expenditure | yes | yes | no |
| Housing | yes | yes | no |
| Sanitation and water | yes | yes | no |
| Access to facilities | yes | dropped | no |
| Shocks | yes | yes | no |
| Participation in social safety net programs | yes | yes | no |
| Other household income | yes | yes | no |
| Infant and young child feeding: knowledge | yes | yes | yes |
| Spousal communication and decisionmaking | yes | yes | yes |
| Parental information | yes | dropped | yes |
| Financial literacy, time preferences, and agency | yes | yes | yes |
| Women’s Empowerment in Agriculture Index | WEAI | pro-WEAI | yes |
|  |  |  | yes |
| Participation in ANGeL (training attendance, training facilities, training delivery, perceptions about training) | no | yes | yes |
| Knowledge obtained from training (agriculture, fish culture, livestock, nutrition BCC, gender sensitization) | no | yes | yes |
| Attitudes towards IPV | yes | Included in pro-WEAI | yes |

Female questionnaire

|  | Baseline | Endline | Included in male questionnaire |
| --- | --- | --- | --- |
| Food consumption and food security | yes | yes | no |
| Anthropometry, health, illness | yes | yes | no |
| 24- hour recall of food intake | yes | yes | no |
| Household food habits | yes | yes | no |
| Infant and young child feeding (IYCF) and use of micronutrient supplements | yes | yes | no |
| ICYF knowledge | yes | yes | yes |
| Health status of children <2 | yes | yes | no |
| Child illness and use of health services | yes | yes | no |
| Nutrition-related prenatal care of youngest child | yes | yes | no |
| Exposure to nutrition information from health worker, community groups, media | yes | yes | no |
| Access to and use of community clinics | yes | yes | no |
| Maternal nutrition knowledge | yes | yes | no |
| Communication and decisionmaking | yes | yes | yes |
| Mobility |  | Included in pro-WEAI | no |
| Wife’s assets at marriage | yes | dropped | no |
| Parental information | yes | dropped | yes |
| Purdah | yes | yes | no |
| Financial literacy, time preferences, agency | yes | Yes | yes |
| Women’s Empowerment in Agriculture Index | WEAI | Pro-WEAI | yes |
| Social desirability bias | no | yes | no |
| Participation in ANGeL (training attendance, training facilities, training delivery, perceptions about training) | no | yes | yes |
| Knowledge obtained from training (agriculture, fish culture, livestock, nutrition BCC, gender sensitization) | no | yes | yes |
| Attitudes towards IPV | yes | Included in pro-WEAI | yes |
| Experience of IPV (following WHO protocol) | yes | Yes | no |
